# Strategies and Technologies for Comprehensive Characterization of Hydrogel-Based Materials in the Biomedical Field

**DOI:** 10.3390/polym18151805

**Published:** 2026-07-23

**Authors:** Sabrina Caria, Jessica Petiti, Federico Picollo, Carla Divieto

**Affiliations:** 1 Physics Department, Università di Torino, 10125 Turin, Italy; federico.picollo@unito.it; 2 Division of Advanced Materials Metrology and Life Sciences, Istituto Nazionale di Ricerca Metrologica (INRIM), 10135 Turin, Italy; j.petiti@inrim.it (J.P.); c.divieto@inrim.it (C.D.); 3 Istituto Nazionale di Fisica Nucleare (INFN) Turin Section, 10125 Turin, Italy

**Keywords:** hydrogel, characterization methods, characterization techniques, biomedical applications, hydrogel properties

## Abstract

Hydrogels represent a unique class of materials characterized by their three-dimensional, hydrophilic polymer networks, which enable them to absorb and retain large quantities of aqueous solutions. While the literature is abundant with excellent works on the classification, preparation, and applications of hydrogels, a notable lack remains in comprehensive studies focused on their characterization. This gap contributes to heterogeneous results and conflicting fields. In this context, this review aims to provide a detailed and systematic overview of hydrogel characterization techniques. It begins with an exploration of physicochemical, morphological, and mechanical properties, followed by an examination of distinctive hydrogel features, biocompatibility, and application-based properties, particularly for testing applications within the biomedical field. By highlighting the significance of various characterization methods, this review seeks to enhance the understanding of hydrogels and guide future research efforts, ultimately facilitating the development of more effective hydrogel-based materials for several applications.

## 1. Introduction

Hydrogels are a unique class of materials characterized by a three-dimensional structure formed through the cross-linking of water-soluble polymers. The cross-linking process creates a stable and durable polymer framework that is water-insoluble, enabling it to retain significant amounts of liquids without compromising the structural integrity. Hydrogels can be described as biphasic materials, consisting of a porous and permeable solid backbone that traps water or other interstitial fluids, creating a liquid content of at least 10%. The ability to absorb and retain water is particularly of interest for applications within the biomedical field of research; indeed, they can effectively replicate the aqueous environment of animal tissue, supporting the physiological functions of cells [1,2,3].

The first appearance of the term “hydrogel” was in 1894, intending to describe a colloid made of inorganic salts [4]. While in 1949, the first biomedical application of hydrogel was reported, Ivalon, a poly(vinyl alcohol) (PVA) cross-linked with formaldehyde used for implants. In the 1960s, Otto Wichterle contributed to the modern understanding of hydrogels due to his work on synthetic materials for medical applications, focusing on biocompatibility and mechanical properties that can mimic living tissues. The hydrogel market was boosted by the introduction of poly(2-hydroxyethyl methacrylate) (pHEMA), becoming a milestone for contemporary hydrogels [5]. Over the following decades, advancements in biocompatibility and oxygen permeability were reached for chemically cross-linked hydrogels. Summarizing the historical overview of hydrogels, three generations of hydrogels can be identified: first-generation hydrogels that include polymers of alkene monomers and cellulose-based hydrogels; second-generation hydrogels that are primarily poly(ethylene glycol) (PEG)/polyester block copolymers that respond to environmental stimuli; and third-generation hydrogels that focus on advanced cross-linking methods to enhance specific properties [4,6]. Recent developments of hydrogels are focused on new synthesis and cross-linking strategies, “smart” hydrogels able to respond to environmental stimuli, together with the incorporation of nanoparticles. These developments have led to the development of hydrogels with improved physicochemical and biological characteristics, broadening their potential applications [7]. Currently, over a hundred medical products utilizing hydrogels have been successfully developed for applications in aesthetics, tissue repair, wound healing, and more [8].

Comprehensive reviews on the classification, preparation, and possible applications of hydrogels are widely present in the literature. Hydrogels can be classified based on their sources—natural, synthetic, or hybrid; their polymeric composition—homopolymeric, copolymeric, or multipolymer interpenetrating polymeric hydrogel; their structure—amorphous, semicrystalline, or crystalline; the type of cross-linking—physical, covalent, or ionic; the gelation process—photo-initiated, thermal, or chemical; the physical appearance—bulk, film, or microsphere; the responses given—chemical, physical or biochemical; or their network electrical charge—non-ionic, ionic, amphoteric electrolyte, or zwitterionic [9,10,11,12,13,14].

Hydrogels can be prepared through either physical or chemical cross-linking approaches. Physical cross-linking approaches included ionic interactions, hydrogen bonds, crystallization, hydrophobic interactions, and protein interactions. Chemical cross-linking approaches included free-radical polymerization, non-free-radical polymerization, enzymatic reactions, solution polymerization, suspension polymerization, grafting to support, polymerization by irradiation, semi-interpenetrating network, or complex coacervation [9,15,16,17,18,19,20,21].

The possible applications of hydrogels encompass numerous fields of research, including hygienic products, agriculture, sealing, artificial snow, food additives, barrier materials to regulate biological adhesions, biosensors, and biomedical applications such as 3D cell culture, tissue engineering, and regenerative medicines, drug delivery systems, and wound dressing [7,9,22,23,24,25,26,27,28,29,30].

Existing literature often addresses characterization within these broader topics, resulting in either generalized overviews [31] or deep analysis limited to specific properties [32]. Consequently, a comprehensive and detailed technical map of all available characterization techniques is currently missing. This review addresses this gap by providing a detailed and structured assessment of the analytical tools used in the hydrogel research domain. This work is organized into distinct sections that focus on key aspects of hydrogel characterization, with the final aim of addressing this gap. Each section is composed of an introduction to the relevant characterization techniques, outlining their specific purposes, advantages, and limitations within hydrogel characterization. This structured approach not only highlights the importance of each technique in understanding hydrogel properties but also allows researchers to investigate which applications the material is most suitable for. By presenting a detailed overview of these characterization methods, this review aims to serve as a valuable resource for researchers in the field, facilitating a deeper understanding of hydrogel properties and their implications for various biomedical applications.

## 2. Physico-Chemical Characterization

The distinctive physical and chemical traits of a material are defined as its physicochemical properties. These properties influence interactions, stability, and functionality across different applications and under varying conditions. The assessment of physicochemical properties is essential to predict performance, processing capabilities, and suitability for specific uses.

**Figure 1:**
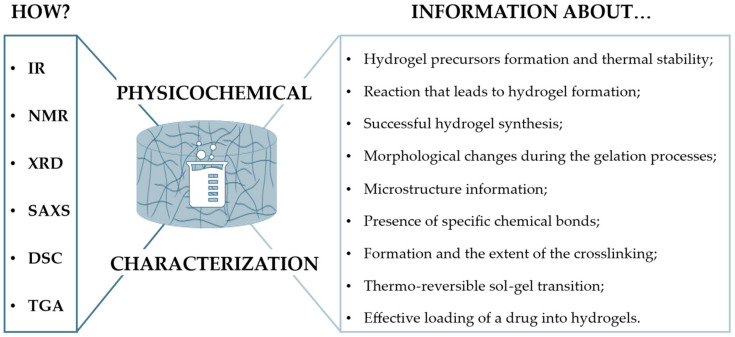
Schematic representation of hydrogel physicochemical characterization techniques, illustrating the various methods employed and the information that can be obtained.

In the context of hydrogels, physicochemical characterization can be performed with various characterization techniques, including infrared spectroscopy (IR), nuclear magnetic resonance (NMR), small-angle X-ray scattering analysis (SAXS), X-ray diffraction analysis (XRD), differential scanning calorimetry analysis (DSC), and thermal gravimetric analysis (TGA). These techniques can be utilized to gather essential information about hydrogel precursors, the reaction that leads to the successful hydrogel formation, possible morphological changes during the gelation processes and microstructure information, properties related to the presence of specific chemical bonds, the formation and the extent of the cross-linking, the thermo-reversible gelation of some hydrogels, and the effective loading of a drug into hydrogels, as shown in Figure 1.

### 2.1. Infrared Spectroscopy (IR)

IR measures the interaction of infrared radiation with matter. Particularly, this spectroscopic technique can detect the vibration of functional groups within a molecule that induces a change in the dipole moment [28,30,33,34]. Fluctuations in the dipole moment interact with the IR radiation, resulting in absorption when the radiation frequency matches the characteristic vibrational frequency of the molecules. Indeed, the IR spectra are plots of measured infrared intensity versus wavelength—or frequency—of light, which are distinctive and characteristic of the sample in question [28,30,33,34].

IR ensures high scan speed, rapid data collection, high resolution, the possibility of analyzing a wide range of organic and inorganic compounds in various phases, the ability to provide extensive structural information, and non-destructive sample analysis. However, IR limitations include the ineffectiveness for samples containing water, the complexity of spectrum interpretation requiring significant experience, the need for complementary analysis in many cases, and limitations in quantitative results at very high or low concentrations [35]. While the strong IR absorption of water represents a fundamental challenge for the analysis of hydrated hydrogels, this limitation is typically mitigated through specific experimental strategies. Common workarounds include the use of attenuated total reflection-FTIR to minimize the optical path length [36] or sample dehydration prior to analysis to remove the liquid content [37]. To ensure the reproducibility of these approaches, standard protocols including American Society for Testing and Materials (ASTM) E1252 [38] standard provides established guidelines for qualitative infrared analysis of polymers and composite materials. This framework is particularly useful when optimizing the contact pressure in ATR crystals or validating alternative spectral subtraction routines for highly hydrated networks. However, since high water content remains a critical limiting factor for detecting subtle chemical interactions, implementing the quantitative criteria outlined in ASTM E168 [39] is suggested to isolate overlapping spectral bands and accurately resolve cross-linking kinetics.

Within the hydrogel framework, IR spectra can be employed to analyze the formation and the purity of hydrogel precursors. Kang et al. analyzed the FTIR spectra of the precursors needed to synthesize a hydrogel based on modified acrylic dextran. The spectra confirmed the formation of both the precursors—2-[(1-oxo-2-propen-1-yl)oxy]ethyl 1H-imidazole-1-carboxylate and poly(2-dextranoyl)-1-carbonyl-oxy-1,2-ethanediyl-acrylate—due to the presence of the characteristic peaks [40]. Similarly, Muthumari et al. confirmed the formation of extracted galactoxyloglucan (GXG) through the comparison of the FTIR spectrum with FTIR spectra of standard sugar units and sodium alginate. The presence of peaks linked to structural components of both reagents confirmed the successful synthesis of the hydrogel precursor [41].

The successful synthesis of hydrogels can be monitored with IR through the identification of characteristic peaks related to chemical bonds that should be formed during the synthesis reaction. In the study done by Mansur et al., the presence in the FTIR spectra of hydroxyl and acetate groups together with the reduction in O-H peak intensity upon cross-linking with glutaraldehyde suggested the formation of acetal bridges and the successful formation of the poly (vinyl alcohol) (PVA) hydrogel structure [42]. In another study performed by Verma et al. FTIR spectra highlighted peaks related to typical stretching vibrations of -CH-O-CH_2_ and C-O in -COOH groups, together with the presence of a peak related to the C = C double bond. This information confirms the formation of carboxymethyl cellulose-cross-linked-N’N-dimethyl acrylamide/acrylic acid hydrogel [43].

Some hydrogel properties are linked to the presence or the absence of specific functional groups within their network. IR analysis can be employed to predict hydrogel behavior based on the presence or absence of characteristic peaks. For instance, Rihawy et al. associated the enhanced hydrophilicity properties with improved water absorption and hydrophilic drug retention ability with the presence of carbonyl and hydroxyl groups in the IR spectra [44]. Similarly, the detection of amide characteristic peaks in the alginate-gentamicin hydrogel can explain its high stability in different solvents due to the formation of a 3D network crosslinked by amide bonds, as suggested by Kondaveeti et al. [45].

Moreover, IR is a useful tool for monitoring the hydrogel cross-linking reactions as reported by Wang et al., where the FTIR spectrum was used to assess the presence of crosslinked gelatin by oxidized starch and the presence of crosslinked gelatin in alginate/gelatin blended hydrogel [46].

The driving force behind hydrogel formation can be understood by examining the interactions among its constituent components using IR. For instance, the FTIR results highlighted by Tang et al. demonstrated that the creation of a polypseudorotaxane hydrogel is primarily facilitated by hydrogen bonding between adjacent α-Cyclodextrin (αCD) molecules, along with host-guest interactions between PEG chains and αCD [47]. Similarly, the attenuated total reflection-FTIR findings reported by Long et al. indicated that the thermo-reversible gelation mechanism of the chitosan-pectin hydrogel is due to hydrogen bonds forming between the -OH groups of chitosan and pectin at lower temperatures, which break as the temperature increases [48].

Hydrogels are increasingly recognized as effective drug delivery platforms, necessitating verification of the drug’s incorporation within their structures. IR analysis is a valuable tool for assessing both the successful integration of drugs into hydrogels and the potential interactions between the drug and the hydrogel matrix. In the study by Chatterjee et al., gallic acid was loaded into Pluronic F-127 (PF127)/N,N,N-trimethyl chitosan (TMC)/PEG-hyaluronic acid (HA) and PF127 hydrogels. The FTIR spectrum revealed a broad peak at the same wavenumber in both hydrogel formulations, indicating that gallic acid was efficiently incorporated within the gel structures [49]. Similarly, Alsakhawy et al. performed IR analysis on naringin (NAR) loaded into an Arabic gum/pectin hydrogel. The presence of characteristic NAR peaks confirmed its integration within the hydrogel, although no significant interactions between NAR and the hydrogel matrix were detected [50].

### 2.2. Nuclear Magnetic Resonance (NMR)

NMR is an analytical technique used to determine molecular structures. Its working principle relies on the magnetic properties of certain atomic nuclei—such as hydrogen-1 (^1^H) and carbon-13 (^13^C)—which have magnetic moments due to their spin. When placed in a magnetic field, these nuclei can align either with or against the field, creating two distinct energy states. During NMR experiments, the sample is exposed to a strong external magnetic field that causes the nuclear spins to align, resulting in a net magnetization along the field’s direction. An RF pulse then excites the nuclei to a higher energy state. When the pulse is turned off, the nuclei relax back to equilibrium, releasing RF signals that are detected by the NMR instrument. The frequency of these signals depends on the electronic environment surrounding the nuclei, causing a chemical shift that provides insights into the molecular structure, including atom types and connectivity. These signals are transformed into a spectrum via Fourier transformation, allowing detailed structural analysis of the molecule [51].

NMR is a highly valuable analytical tool renowned for its ability to analyze biological samples, such as algae, and provide detailed structural information. This non-destructive technique requires minimal sample preparation and significantly reduces analysis time. NMR is automated, efficient, and utilizes very small sample volumes. However, there are notable limitations that can impede its effectiveness. A major concern is the natural abundance of isotopes required for analysis; for example, the abundance of ^13^C is approximately 11%, while oxygen-17 (^17^O) is a mere 0.035%. This low abundance complicates measurements for certain elements. Furthermore, some nuclei exhibit low magnetic moments, adversely affecting the sensitivity of NMR instruments. When the magnetic moment is too weak, achieving spectra with adequate peak intensity becomes difficult, complicating signal detection and interpretation [52]. In this scenario, implementing the experimental and calibration guidelines specified in ASTM E386 [53] aids in maximizing instrument sensitivity, thereby enhancing the reliability of quantitative NMR characterizations on soft networks.

NMR represents a pivotal method for investigating the efficacy of hydrogel precursor synthesis as well as the hydrogels themselves. Kang et al. demonstrated this concept by using ^1^H-NMR spectroscopy to analyze the synthesized poly(2-dextranoyl)-1-carbonyl-oxy-1,2-ethanediyl-acrylate (PED), confirming its presence and thereby validating the synthesis of one hydrogel precursor [40]. Similarly, Huang et al. confirmed the successful synthesis of gelatin methacryloyl (GelMA) through ^1^H-NMR analysis. Their findings showed a significant reduction in the amine groups (-NH_2_) of gelatin, indicating a high degree of substitution in GelMA, along with the identification of new peaks associated with the methacryloyl moiety, confirming the incorporation of this functional group into gelatin [54]. Additionally, ^1^H-NMR was employed to verify the composition of two co-polypeptides in another study [55].

The ^1^H-NMR spectra can also provide insights into the chemical reactions leading to hydrogel formation. Several studies have leveraged ^1^H-NMR spectroscopy for this purpose. For instance, specific peaks associated with the N-methylation process were detected, confirming the successful synthesis of TMC. Moreover, the reaction between HA and methoxy-polyethylene glycol amine indicated the successful formation of PEG-HA through an amide coupling reaction, as evidenced by the emergence of new peaks corresponding to ethylene and -NH_2_ [49].

Comparative analysis of NMR spectra between the precursors and the final hydrogel can help determine the success of synthesis. In a study by Ding et al., a comparison of the ^1^H-NMR spectra of chitosan and sialylated-chitosan hydrogel showed that the absence of several peaks in the spectrum of raw chitosan, compared to the chitosan and sialylated-chitosan conjugate, provided strong evidence for the successful grafting of sialic acid (SA) onto chitosan [56].

Furthermore, NMR can assess the extent of cross-linking in superabsorbent networks, investigate interactions with additives, determine solid-state structural properties and packing configurations, characterize various polymorphic forms, and evaluate conformational changes that influence gelation properties [57].

### 2.3. Small-Angle X-Ray Scattering Analysis (SAXS)

SAXS is an analytical technique within the family of X-ray scattering methods. It measures the intensity of X-rays scattered by a sample as a function of the scattering angle, typically covering angles up to 1 degree. SAXS is employed for analyzing sub-nanometer-sized structures, providing insights into nanoparticle size distribution, the dimensions and shapes of macromolecules, and pore sizes within materials. The sample is scanned with a SAXS goniometer, which measures angles and permits precise rotation of the sample. When X-rays are directed at the material, they scatter off the electrons within it, producing a scattering intensity that is plotted against the 2θ angle. The resulting scattering pattern can reveal significant information about the internal structure of the material, including atomic arrangement and the presence of nanostructures [58].

SAXS presents several advantages, including minimal preparation—typically requiring only filtration before loading the sample into a measurement capillary—and rapid measurement times, generally between 1 to 90 s. It collects data in conditions that closely mirror the natural state of the samples, making it particularly suitable for those that cannot be prepared using other techniques. This method is effective for studying proteins with flexible linkers and intrinsically disordered proteins, and it integrates well with data from complementary structural methods. Furthermore, the availability of international guidelines, e.g., International Standards Organization (ISO) 17867 [59], can enhance the methodological robustness of the technique by providing standardized frameworks for calibration and data processing. Conversely, SAXS also has limitations. The spatial resolution of models derived from SAXS is generally limited to around 2 nm or higher, which can restrict detailed structural analysis. Furthermore, isotropic scattering caused by random particle orientation complicates data interpretation. Achieving optimal contrast is often challenging due to similarities in electron density between biological samples and their buffers. The presence of electron-rich substances in buffers can interfere with measurements, and SAXS frequently depends on prior information from other techniques, which may influence its effectiveness as a standalone method in some situations [60].

SAXS is highly applicable within the hydrogel framework, particularly for observing morphological changes during the gelation process. This can be achieved by either acquiring a series of SAXS profiles as gelation occurs or by comparing SAXS profiles of the hydrogel with those of its precursors. For instance, Wu et al. conducted in situ SAXS analyses on hydrogels, demonstrating that a gradual branching of the network structure characterizes the gelation process [55]. Another study compared SAXS spectra of GelMA–poly(*N*-isopropylacrylamide) (PNIPAM) hydrogel and GelMA solution, revealing that the incorporation of NIPAM results in a new and larger structure [54].

SAXS also plays a significant role in analyzing hydrogel microstructures. Chatterjee et al. utilized SAXS to investigate the presence of frozen inhomogeneities in a gallic acid-loaded hydrogel system (PF127/TMC/PEG-HA), which were attributed to highly electron-dense crystallized regions resulting from inherent network defects in the hydrogels [49]. In another study, SAXS was employed to examine the microstructure of Chitosan–Catechol (CC) hydrogels with varying solid contents. The findings indicated that the hydrogels maintained a stable structure over time, with one formulation being particularly sensitive to strain changes and requiring a longer time to return to a steady state. Additionally, in situ SAXS analysis combined with rheology was used to study microstructural changes during hydrogel deformation. The results suggested that at low strains, the hydrogel network is stable, but it is gradually compromised as strain increases due to bond rupture [61].

Furthermore, SAXS can monitor drug loading into hydrogels by analyzing microstructural changes. For instance, Kulkarni et al. demonstrated that SAXS analysis confirmed the drug loading into lipid particles, causing a shift in their nanostructure from a bicontinuous cubic phase to a hexagonal phase [62].

### 2.4. X-Ray Diffraction Analysis (XRD)

XRD is a non-destructive technique employed to determine the composition and crystalline structure of a sample. It measures the intensity of X-rays diffracted as a function of the rotation angle (2θ), providing critical insights into various physical properties, including phase chemical composition, crystal structure, crystal orientation, crystallite size, lattice strain, preferred orientation, and layer thickness across powder, solid, and liquid samples [63].

In XRD analysis, X-ray beams are directed at the sample, leading to interactions with its atoms, which cause the X-rays to “bounce” off at specific angles known as the angle of diffraction (θ). This angle is analyzed using Bragg’s Law, reported in Equation (1):

sinθ = nλ/2d(1)

XRD is a rapid and powerful technique for identifying unknown minerals, typically providing clear results with minimal sample preparation. In addition, compliance with ASTM E915 [64] criteria could improve the accuracy of phase identification and crystallite structural data. While it is widely accessible and easy to interpret, it works best for homogeneous, single-phase materials and requires a standard reference file. The technique necessitates a minimum sample mass of tenths of a gram, with a detection limit of around 2% for mixed samples. Moreover, indexing patterns for non-isometric crystal systems can be complex, and peak overlap may occur, especially at higher angles [65].

The comparative analysis of XRD patterns between hydrogels and their precursors provides insights into the interactions occurring during their formation. For example, a characteristic peak indicative of a channel-type crystalline structure was observed in an inclusion complex, confirming the incorporation of PEG chains from Tween 80 into the αCD cavity, which formed a necklace-like polypseudorotaxane structure [47].

XRD has been extensively employed to investigate the crystallinity of hydrogels. For instance, a study verified the crystallinity of a hydrogel formulated from modified acrylic dextran, with the crystallinity arising from the partial alignment of molecules facilitated by amphiphilic intermolecular interactions [40].

Additionally, XRD can assess the degree of microstructure assembly within hydrogels. Bardajee et al. determined the porosity of a salep-based hydrogel by analyzing interchain spacing, revealing longer interchain distances, increased flexibility, and greater porosity compared to conventional salep sources [66].

Furthermore, XRD facilitates the investigation of element incorporation into hydrogel matrices. Verma et al. demonstrated the successful integration of boron nitride nanosheets (BNNSs) into a carboxymethyl cellulose-cross-linked-N’N-dimethyl acrylamide/acrylic acid (NaCMC-cl-DMAA/AAc) hydrogel. A peak in the XRD pattern, characteristic of BNNSs, was observed, while this peak was not present in the XRD profile of the NaCMC-cl-DMAA/AAc hydrogel, indicating the incorporation of the nanosheets [43].

### 2.5. Differential Scanning Calorimetry Analysis (DSC)

DSC is a key technique in thermal analysis that measures the heat absorbed or released by a sample as a function of temperature during phase transitions or chemical reactions. The thermal behavior of materials—including melting points, crystallization, glass transition temperature (T_g_), specific heat capacity, cure processes, oxidation behavior, and aspects related to material composition, purity, and crystallinity—can be assessed using the DSC instrument [67,68,69].

In a DSC experiment, a sample is subjected to a controlled temperature change while continuously monitoring the heat flow to observe the various thermal transitions. As the temperature increases, DSC measures the difference in heat flow between the sample and a reference material, allowing for the direct assessment of various thermal properties. The range of analyzable samples is extensive, including biomolecules such as proteins and nucleic acids, as well as nanomaterials and polymers. DSC offers several advantages, including the ability to provide precise thermodynamic data and insights into molecular stability and interactions. However, it necessitates relatively pure samples, and challenges may arise from factors such as sample size and thermal history [67,68,69].

DSC analysis can be utilized to explore the interactions between the polymers in hydrogels and their cross-linkers. For instance, a study by Alsakhawy et al. indicated that the DSC thermograms of hydrogels formed through physical interactions displayed characteristics similar to those of their precursors. In contrast, hydrogels prepared via electrostatic interactions showed modifications in their thermograms [50]. To address this latter constraint, the implementation of standardized protocols, e.g., ASTM D3418 [70], might enhance the data reproducibility and robustness through the introduction of preliminary heating cycles designed to erase the material’s previous thermal and mechanical history.

DSC is also a valuable tool for investigating drug-hydrogel interactions by comparing the DSC thermograms of the free drug and the drug-loaded hydrogel. One study examined a chitosan-pectin hydrogel loaded with lidocaine hydrochloride (LDC), revealing a bimodal crystalline melting transition and a shift in the melting point relative to the thermogram of LDC alone. These findings suggest that recrystallization occurs alongside interactions between the drug and the functional groups of the polymers during the incorporation of LDC into the hydrogel [48]. Furthermore, another investigation reported the disappearance of characteristic endothermic and exothermic peaks associated with both enoxaparin and one of the hydrogel precursors—αCD—further confirming the presence of interactions between the drug and the hydrogel [47].

Additionally, DSC thermograms are effective for studying the thermo-reversible gelation of specific hydrogels. Iijima et al. reviewed the characteristics of cold-set gels, which form upon cooling an aqueous solution, and heat-set gels, which are generated by heating an aqueous solution. Their study also evaluated the thermal behavior of a combination of these two polysaccharides using DSC [71].

### 2.6. Thermal Gravimetric Analysis (TGA)

TGA is utilized to quantify weight loss as a function of temperature variations of sample. TGA measures the mass of a sample while subjected to heating in an inert gas atmosphere. Throughout the analysis, continuous weighing enables the observation of mass changes associated with thermal reactions, which often result in the release of gaseous byproducts. The effective removal of these gases is essential, as it allows for the precise recording of changes in the remaining mass of the sample. TGA provides valuable insights into both physical phenomena—such as absorption, adsorption, and desorption—and chemical processes, including chemisorption, thermal decomposition, and solid–gas reactions [72]. This technique requires only small sample sizes—typically less than 20 mg—while yielding high precision and repeatability. Additionally, it facilitates the estimation of crucial kinetic parameters, which are instrumental in understanding reaction mechanisms pertinent to pyrolysis processes. In contrast, TGA is confined to processes that involve mass changes and is unable to assess thermal events such as melting or phase transitions. Furthermore, TGA typically generates minimal residual product, which can hinder its practicality for yielding pyrolysis products. The method is inherently destructive to the sample and sensitive to experimental conditions, necessitating careful consideration in its selection to ensure the consistency and reliability of results. Moreover, interpreting data from integrated analyses can introduce complexity, requiring a nuanced understanding of the underlying interactions being studied [73]. In this context, the accuracy of mass-loss quantification can be improved following ASTM E1131 [74] standard guidelines, facilitating clearer data interpretation across complex polymer networks.

TGA can be utilized to investigate the thermal stability of hydrogels prepared with various formulations, where an increase in thermal stability typically correlates with enhanced cross-linking stability. For instance, a study examining gelatin/carboxymethyl cellulose hydrogels at different blend ratios identified that the cross-linked hydrogel with a 10:1 blend ratio exhibited improved thermal stability [75]. In another study, TGA was employed to evaluate the effects of incorporating BNNSs into the NaCMC-cl-DMAA/AAc hydrogel formulation, revealing that BNNSs incorporation increases the thermal stability of the overall hydrogel [43].

TGA thermograms can serve to assess the thermal stability of hydrogel precursors and hydrogels themselves, offering insights into the efficiency of cross-linking. For example, one study highlighted significant weight loss differences between a precursor and its corresponding hydrogel, indicating the presence of photocured residue attributed to cross-linkable functional groups [40]. In another study, the TGA thermograms of GXG and SA were compared with those of the blended GXG-SA hydrogel. The results suggested a reduction in weight loss for the blended hydrogel compared to the individual components, indicating enhanced thermal stability due to cross-linking and ionic interactions [41]. Additionally, another study demonstrated that the thermal stability of hydrogel markedly increased compared to its precursors, further supporting the positive effects of the synthesis process [66].

## 3. Morphological Characterization

**Figure 2:**
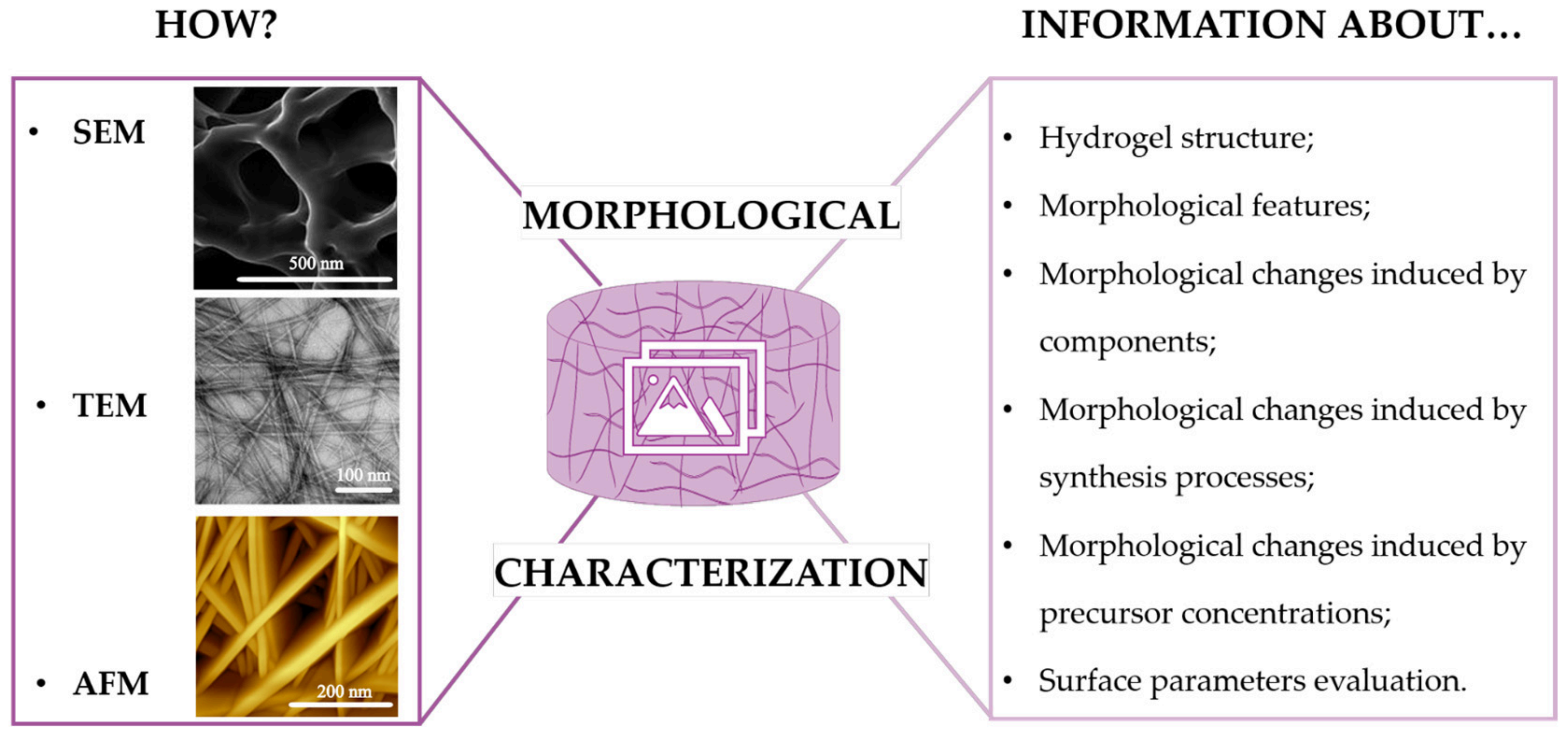
Schematic representation of hydrogel morphological characterization techniques, illustrating the various methods employed and the information that can be obtained. The SEM image is adapted from [77] (MDPI, 2025), TEM image is adapted from [78] (MDPI, 2024), and AFM image is adapted from [79] (MDPI, 2022).

The morphological and microstructure features of materials for understanding the structure and form of materials at various scales, from macroscopic to nanoscale dimensions, are a fundamental aspect of the field of materials science research. This field encompasses the analysis of physical and chemical characteristics, including the size, shape, arrangement, and distribution of constituent phases. Morphological properties, such as particle size, shape, and surface topography, significantly influence key material characteristics like ductility, durability, and strength. Likewise, microstructural features, including inclusions, cracks, and pores, play a crucial role in determining material behavior under varying conditions. The relationship between morphology and microstructure is essential for predicting material performance and optimizing design and manufacturing processes [76]. These techniques offer crucial insights into the hydrogel’s surface topography and internal structure, revealing morphological variations resulting from specific components and formation processes. Furthermore, surface modifications and chemical agents can profoundly influence the morphology of hydrogels, affecting essential properties such as swelling behavior and drug loading capacity, as graphically represented in Figure 2. By examining hydrogels with different precursor concentrations, researchers can evaluate how these variables impact overall morphology, facilitating the selection of the most appropriate hydrogels for targeted applications.

### 3.1. Scanning Electron Microscopy (SEM)

SEM is a powerful imaging technique used to investigate the surface characteristics of materials at micro- and nanoscale resolutions. In SEM, a focused electron beam, with acceleration voltages up to 30 kV, is directed onto the specimen, generating various signals upon interaction with the sample. These signals, which include backscattered and secondary electrons, are collected by detectors to create detailed images of the specimen’s surface. The main types of scattering include elastic scattering, which provides compositional and topographic information through backscattered electrons (BSE), and inelastic scattering, which generates secondary electrons (SE) with energies less than 50 eV. SE are particularly valuable for high-resolution topographic imaging, as they originate from shallow depths near the surface. For non-conductive samples, a thin conductive layer (1–5 nm) of materials like gold or carbon is typically applied to prevent charging effects and enhance image quality. SEM offers numerous advantages, including detailed three-dimensional imaging and versatility through various detectors like SE imaging, BSE imaging, and energy-dispersive X-ray spectroscopy (EDS). Additionally, advances in computer technology have made SEM user-friendly and efficient. However, SEM imaging is limited to solid, inorganic samples that can fit within the vacuum chamber. To address this, techniques such as sputter coating and operation in low-vacuum or Environmental SEM (ESEM) modes have been developed [80,81,82,83]. Low-vacuum SEM allows for the examination of uncoated samples, particularly delicate materials that cannot withstand high vacuum. This method enables the direct imaging of biological specimens and other soft materials without extensive preparatory processes, though it trades off some resolution due to gas presence affecting the electron beam. ESEM, on the other hand, enables imaging under conditions that closely resemble natural environments, requiring minimal sample preparation. Operating at higher pressures (up to approximately 67 mbar), ESEM can work with moist samples and electrically insulating materials, as gas molecules help neutralize charging effects. While this technique may exhibit reduced spatial resolution due to electron scattering, it is particularly advantageous for studying delicate or irreplaceable samples without conventional preparation methods [84].

Within hydrogel characterization, SEM can be particularly useful to examine morphological features. One study employed SEM to investigate the morphology of a nanoporous hydrogel, revealing that the gas-blowing method used in its synthesis produced a honeycomb-like structure with aligned channels and varying pore diameters. Bardajee et al. highlighted the relevance of these findings in the identification of the most suitable application of this hydrogel; indeed, the nanoporous surface is particularly adept at absorbing or trapping molecules and ions of different sizes and charges [66].

Another study compared the microstructure of alginate-polypyrrole (Alg + PPy) and alginate/gelatin-polypyrrole (Alg/Gel + PPy) hydrogels, revealing a variation in the micro-sized pores, with average diameters of 20 ± 7 μm for Alg + PPy and 18 ± 7 μm for Alg/Gel + PPy. These features render both hydrogels suitable for mesenchymal stem cell (MSC) mitochondria transplantation in regenerative applications [85].

Zhou et al. evaluated the internal structure and pore architecture of alginate hydrogels, both containing and lacking carbon nanoparticles with SEM. Although the incorporation of carbon nanoparticles did not significantly alter pore sizes, an increase in the thickness of the pore walls was observed with higher concentrations of nanoparticles [86].

In another study, the impact of AgNO_3_ concentrations on the distribution and morphology of nano-silver particles within the hydrogel matrix was assessed using SEM. The findings indicated that the hydrogels maintained a highly interconnected porous network, with nano-silver particles uniformly distributed throughout. Notably, the size of the nano-silver particles increased alongside higher concentrations of AgNO_3_ [87].

#### The Effect of Hydrogel Preparation for SEM Analysis

While SEM is a widely used technique for examining the nanoarchitecture of hydrogels, it is important to recognize that the methods of sample preparation can significantly impact the observed structures. Challenges arise in freezing or removing water without disturbing the delicate architecture of hydrogels, and the effects of sample preparation on hydrogel structure have not been thoroughly documented.

A study conducted by McFetridge et al. sought to compare various fixation methods for self-assembling peptide hydrogels to assess the impact of sample preparation on β-peptide hydrogels [88]. The first method employed a traditional SEM approach, which involved chemical fixation, dehydration through a series of ethanol solutions, critical point drying, and gold sputtering. The sample prepared through the standard procedure exhibited minimal alignment and a few large pores. The second method used a standard Cryo-SEM procedure that included high-pressure freezing, freeze-fracture under liquid nitrogen, etching, and gold/palladium sputtering. The Cryo-SEM approach produced highly aligned fibers and a regular mesh-like structure. The third method combined aspects of both approaches by transferring samples from high-pressure freezing to critical point drying through freeze substitution in ethanol. The combined method resulted in some aligned fibers and larger open spaces not observed in the Cryo-SEM samples.

McFetridge et al. demonstrated that the reliability of information regarding hydrogel structure using SEM remains questionable due to its dependence on pre-analysis treatments. While the high-pressure freezing step was successful in preserving fiber alignment and exposing internal structures through freeze-fracture, traditional SEM primarily targets the surface, which may result in the neglect of internal arrangements. Although high-pressure freezing reduced ice crystal artifacts, some artifacts persisted. This study emphasizes the significant impact that various preparation methods have on hydrogel nanoarchitecture, thereby fueling ongoing discussions about enhancing the characterization of hydrogels for different applications [88].

### 3.2. Transmission Electron Microscopy (TEM)

TEM offers the possibility for high-resolution imaging and analysis of materials’ internal structures at the atomic or molecular scale. In TEM, a high-energy electron beam is transmitted through a very thin specimen (less than 100 nanometers), interacting with atomic structures and creating images and diffraction patterns. TEM consists of several key components, including an electron source, magnetic lenses, a specimen holder, and detectors. This setup enables the examination of various samples, such as metals, semiconductors, biological specimens, and nanomaterials. The extensive information obtained encompasses morphology, crystallography, composition, and variations in thickness and density. Moreover, the high resolution allows for detailed analysis of particle shape and size, crystal structures, orientations, defects, and even elemental composition through techniques such as EDX. The limitations of TEM included a limited sampling area, complex 2D interpretation of 3D structures, possible sample damage caused by the electron, and the need for a vacuum environment. Additionally, the preparation of ultra-thin specimens can be challenging and may introduce artifacts [89,90,91]. As noted in the SEM analysis, the sample preparation methods for TEM significantly affect the structure of hydrogels, which can be delicate and difficult to preserve during processes like freezing and dehydration [88].

Several studies utilized TEM for examining the morphological features of hydrogels. For instance, Bardajee et al. utilized TEM to assess the nano-dimensions of pores and voids in a salep-based hydrogel, finding a notable correlation with morphological features observed in SEM images obtained through the gas-blowing technique [66]. In another study, 9-fluorenyl methoxycarbonyl-modified aromatic dipeptide hydrogel morphology was investigated with TEM, revealing a network of nanofibers with diameters of approximately 28 nanometers [92].

The capability of TEM images to elucidate morphological differences arising from various formation processes is further exemplified by the work of Wei et al. Their research demonstrated that nanofiber density in hydrogels synthesized via cross-linking is significantly greater compared to those produced through self-assembly. This underscores the importance of formation methods in influencing the structural characteristics of hydrogels, with implications for their potential applications [93].

### 3.3. Atomic Force Microscopy (AFM)

AFM is a powerful and versatile technique for measuring interactions between atoms at distances ranging from 0.1 to 100 nanometers. AFM allows the imaging and the characterization of surface properties at the nanometer scale, enabling detailed surface analysis at the atomic level across various environments. The fundamental principle of AFM relies on a nanoscale tip attached to a cantilever that acts like a spring. When the tip contacts the surface, the cantilever bends, and this deflection is measured using a laser diode and a split photo-detector. The bending reflects the interaction forces between the tip and the sample, including attractive forces such as van der Waals and electrostatic interactions, as well as repulsive forces from hard sphere repulsion and Pauli exclusion. AFM has been employed to study a wide range of materials, including thin and thick film coatings, composites, glasses, membranes, metals, polymers, and semiconductors. It is particularly useful for analyzing processes such as abrasion, corrosion, etching, and friction. The technique provides atomic-resolution imaging and can measure forces at the nano-Newton level. Key advantages of AFM include the ability to generate 3D surface profiles and preserve sample integrity without requiring special treatments, making it suitable for biological materials. AFM operates in ambient air or liquid environments, allowing for the study of biological macromolecules and live organisms. However, AFM has limitations, including a restricted scanning area, and a maximum height measurement capability of only 10–20 µm. The scanning speed is generally slow, leading to potential thermal drift that can impact accuracy. Additionally, image quality may suffer from hysteresis and crosstalk between axes, necessitating software corrections that can obscure true features. Standard AFM probes may also face challenges with steep structures, requiring specialized cantilevers that can affect resolution and introduce artifacts [94,95,96].

AFM is a valuable technique for examining surface modifications and the effects of chemical agents on materials, which can influence hydrogel properties. For instance, studies have shown that the adsorption of ions can lead to notable changes in surface topography, such as agglomeration of grains and variations in surface features. Increased concentrations of these ions often correlate with heightened surface roughness and complexity [97]. Additionally, AFM can be used to compare different hydrogel materials by assessing surface parameters, aiding in the selection of suitable options for various applications. Surface roughness is particularly important in devices interacting with living systems, as it can significantly affect biological reactivity. Evaluating the roughness of various hydrogel contact lenses has revealed that differences in surface treatment and material composition can impact wettability, surface characteristics, and overall biocompatibility [98].

Moreover, AFM is useful for studying the topographical differences in hydrogels subjected to different processing conditions, highlighting how factors such as precursor concentrations and treatment cycles can alter structural properties. These insights underscore the dependence of hydrogel characteristics on their formulation and processing methods [99].

### 3.4. Comparison Between SEM, TEM, and AFM

SEM, TEM, and AFM are effective techniques used to investigate the morphological features of hydrogels. Each method offers unique insights into hydrogel structure and characteristics. A study by Adhikari et al. sought to leverage the strengths of these imaging techniques by synthesizing short peptide-based hydrogels and conducting a comprehensive morphological analysis using all three methods [100]. The integrated findings from SEM, TEM, and AFM highlighted the structural characteristics of the hydrogels, showcasing both the advantages and limitations of each technique in evaluating hydrogel morphology. All the techniques allowed the collection of information regarding the hydrogel structure and the fiber width. Field emission SEM (FE-SEM) revealed a nanofibrillar network structure responsible for gelation, with fibril widths of 45–70 nm for peptide 1 and 45–90 nm for peptide 2. TEM images showed bundles of fibrous aggregates, with fiber widths ranging from 20–42 nm for peptide 1 and 25–45 nm for peptide 2, while the hydrogel from peptide 2 exhibited a denser cross-linked network with fiber widths of 30–60 nm. AFM analysis of xerogels demonstrated a highly entangled three-dimensional nanofibrous network, with fiber diameters between 48 to 75 nm for peptide 1 and 45–90 nm for peptide 2, indicating significant fiber–fiber interactions. A confocal microscopy study of the hydrated hydrogel revealed the cross-linked fibers’ microstructure, allowing the study of the hydrogel’s behavior in physiological conditions. The uniformity of the supramolecular aggregates across the imaging techniques suggested that the peptide molecules were self-assembled hierarchically to form a one-dimensional supramolecular polymer. The width of the gel fibers being greater than the molecular dimensions of the gelator peptide indicated that multiple molecular chains aggregate to create the nanofibrillar structure [100].

## 4. Mechanical Characterization

Mechanical properties of materials are critical aspects that govern their performance and ability to maintain integrity under various conditions. Key parameters within this realm include strength, which measures a material’s capacity to withstand external forces without fracturing; stiffness, indicating its resistance to deformation under stress; and elasticity, the ability to return to its original shape after deformation. Additional characteristics, such as plasticity, allow for permanent deformation without failure, while ductility enables materials to be drawn into thin wires. In contrast, brittleness describes materials that fracture easily with minimal distortion [101].

Furthermore, malleability refers to a material’s capacity to be shaped into thin sheets without cracking, whereas toughness represents its ability to absorb energy and resist fracture under high-impact loads. The phenomenon of creep occurs when a material subject to constant stress at elevated temperatures undergoes slow, permanent deformation. Additionally, resilience measures a material’s capability to absorb energy from shocks, while fatigue denotes failure under cyclic loading conditions. Finally, hardness is defined as the resistance a material has to penetration by another object [101].

**Figure 3:**
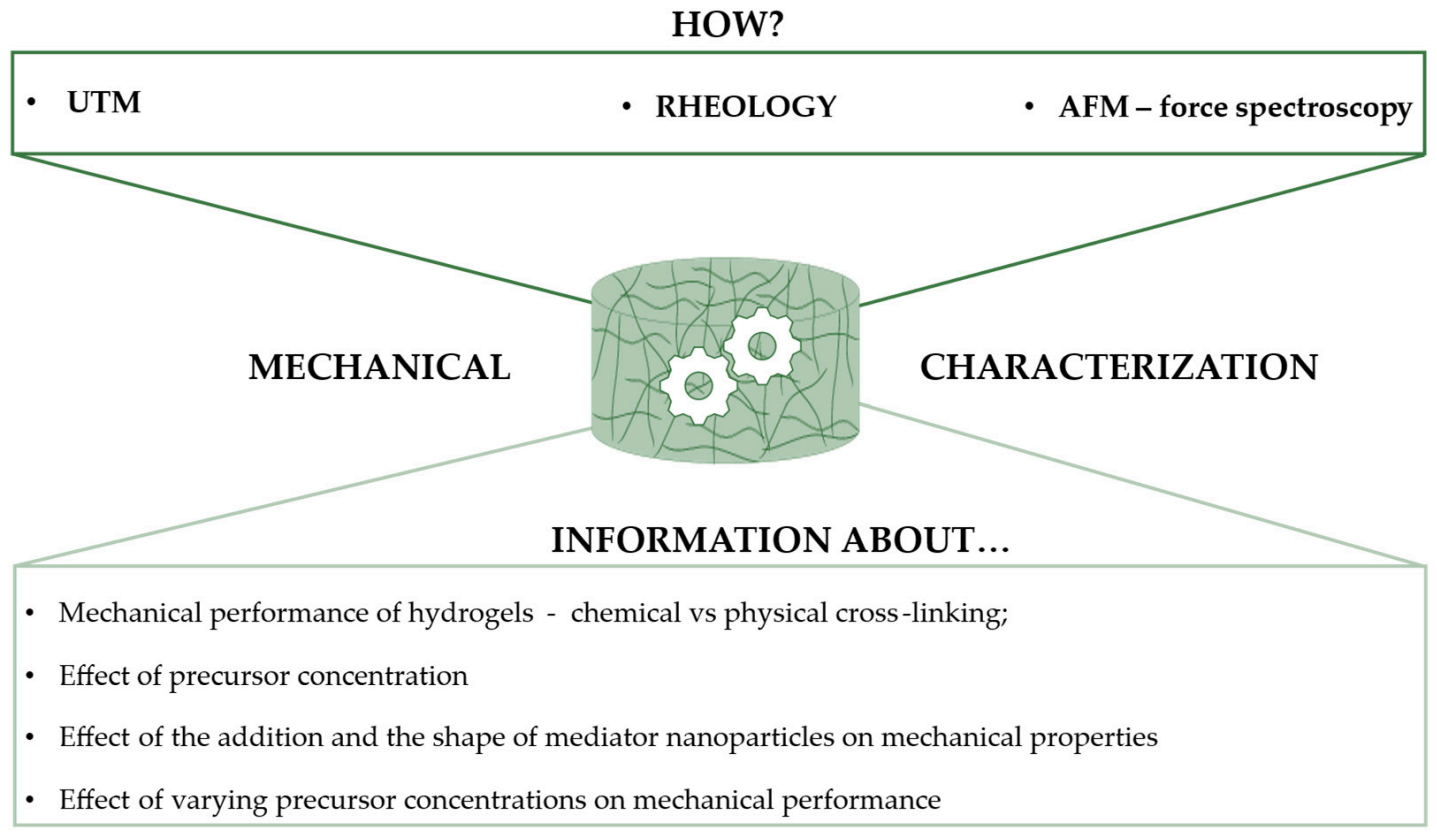
Schematic representation of hydrogel mechanical characterization techniques, illustrating the various methods employed and the information that can be obtained.

To characterize the mechanical properties of hydrogels effectively, researchers utilize various techniques, including universal testing machines (UTM), rheology, and atomic force microscopy (AFM). These methods yield valuable insights into the mechanical performance of hydrogels, especially in distinguishing between those with chemical versus physical cross-linking. They also allow for a thorough evaluation of how precursor concentration and the presence and shape of mediator nanoparticles impact mechanical properties, as illustrated in Figure 3. By investigating these factors, researchers can determine the most suitable hydrogels for specific applications, thereby advancing the understanding and utility of hydrogels across various fields.

### 4.1. Universal Testing Machine (UTM)

UTM, commonly referred to as universal testers or tensometers, are crucial instruments utilized for the mechanical characterization of materials. These multifaceted devices enable the assessment of a wide range of mechanical properties, including tensile and compressive strength, flexural strength, bending, shear, hardness, and torsion. UTMs function by applying controlled mechanical loads to specimens while simultaneously measuring the resulting deformation or displacement, facilitating the determination of vital parameters such as tensile strength, yield strength, modulus of elasticity, and elongation at fracture. The advantages of UTMs include rapid and reliable testing procedures, versatility across multiple testing modes, and compliance with international standards, ensuring consistent and comparable results. However, challenges persist, such as the complexities associated with integrating UTMs into existing workflows, potential limitations in capability for various materials, and difficulties in achieving standardization across global production sites [102,103].

UTMs are particularly effective for analyzing soft materials, such as hydrogels, which typically require a load cell with a force range of 10 to 500 N, given that the Young’s modulus of hydrogels generally resides within the kilopascal range. For instance, research has shown that chemically crosslinked hydrogels demonstrate superior mechanical properties due to the presence of stronger chemical bonds, which offer greater resistance to deformation compared to the hydrogen bonds found in physically crosslinked variants [104].

In a study by Zhang et al., UTM was utilized to explore the impact of varying concentrations of HA on the mechanical properties of PVA/HA composite hydrogels. These composites are regarded as promising candidates for cartilage substitutes in biomedical applications. Their findings indicated that the optimal HA content significantly enhanced stress relaxation properties and impacted on the dissipation capabilities of the hydrogels, making them more suitable for joint protection and cartilage repair [105].

### 4.2. Rheological Analysis

Rheology, the study of the deformation and flow of materials under applied stress, focuses on how materials respond to forces, with ideal solids exhibiting elastic deformation and fluids flowing while dissipating energy as heat. A key concept in rheology is stress, defined as the force exerted per unit area. When forces are applied in opposite directions parallel to a surface, this phenomenon is termed shear stress. Shear stress is crucial in rheological studies because it measures the force required per unit area to initiate fluid flow. Additionally, the shear rate describes the velocity gradient of the fluid, indicating how quickly the fluid moves between parallel plates under shear stress. Rheometers are essential tools for measuring the rheological properties of materials and can be classified into two primary types: controlled-stress rheometers and controlled-strain rheometers. A controlled-stress rheometer applies torque to maintain stress at a predetermined level or to achieve a specific strain. Conversely, a controlled-strain rheometer positions the material between two plates, with the bottom plate moving at a constant speed [106,107,108]. Rheology has several advantages, including its straightforward execution, which allows for easy measurements with simple setups suitable for various applications. It helps in evaluating different material formulations early in the design process, aiding informed decision-making. Additionally, rheology provides key insights into fluid flow characteristics that are important for optimizing processes in industries like oil and gas, food, and pharmaceuticals. However, there are challenges to consider. One major issue is the difficulty in accurately simulating natural reservoir conditions, which can limit the predictive accuracy of rheological models. Furthermore, factors like changes in pressure and temperature during drilling can complicate measurements, and contamination can affect the properties of drilling fluids, making results less consistent and reliable [109].

In accordance with the small-amplitude oscillatory shear protocol for rheological characterization of hydrogels suggested by Zuidema et al. [110], a possible strategy to compensate for the frequency-dependent nature of viscoelastic hydrogels and navigate these characterization challenges is the execution of a sequential operational testing window. This involves loading precursors as liquids at 37 °C, identifying the Linear Viscoelastic Region via sequential time and strain sweeps, and testing within a standardized window of 1 Hz frequency and 5–10% strain. This approach successfully targets the stable elastic plateau while avoiding measurement artifacts [110].

Rheology has emerged as a pivotal tool in the study of hydrogels, particularly concerning their mechanical properties. Recent investigations have underscored the impact of nanoparticle incorporation and morphology on the behavior of these materials. Arno et al. utilized rheological analysis to explore the influence of nanoparticle shape on the mechanical properties of hydrogels, specifically alginate variants. Their results indicate that different nanoparticle shapes significantly affect the mechanical performance of the hydrogels. This research underscores the importance of nanoparticle morphology in customizing the properties of hydrogels for specific applications, revealing that both the type and shape of nanoparticles are critical in determining the overall mechanical characteristics of the hydrogel system [111]. In another study, Zaragoza et al. explored how nanoparticle-mediated physical cross-linking contributes to an increase in the elastic modulus of chemically crosslinked NIPAM-silica nanoparticles hydrogels. The findings suggest that this enhancement in modulus is closely linked to the interactions occurring in the interfacial zone between the nanoparticles and the polymer matrix, indicating that the presence of nanoparticles not only reinforces the hydrogel structure but also alters its mechanical behavior [112].

### 4.3. AFM—Force Spectroscopy Analysis

AFM has revolutionized the investigation of nanomechanical properties in materials, particularly hydrogels, by enabling the quantification of mechanical attributes such as Young’s modulus and adhesion forces through Force-Distance spectroscopy. As the AFM cantilever approaches the sample, it measures the forces involved, producing a force-distance curve that reflects the mechanical characteristics of the materials. A key factor influencing the accuracy of these measurements is the characteristics of the AFM tip. Research has shown that the shape of the AFM tip significantly affects the results obtained through force spectroscopy [113,114,115]. For instance, studies indicate that Young’s modulus values measured with different cantilevers reveal a trend where modulus values decrease with increased indentation depth and can vary notably based on cantilever shape [116]. This variability highlights the importance of selecting the appropriate tip geometry for reliable mechanical characterization. To obtain trustworthy Young’s modulus measurements in hydrogels, achieving a sufficient indentation depth is crucial. This depth ensures measurements reach a plateau region, indicating that the mechanical response of the hydrogel has stabilized and provides a more accurate representation of its elastic properties. Measurements that do not reach this depth may be influenced by surface effects or artifacts, leading to potentially misleading conclusions about the material’s mechanical behavior [117].

A comprehensive review of the elastic properties of various hydrogels, including those made from materials like alginate, HA, keratin-α, gelMA, and polyacrylamide gels, underscores the importance of material selection and concentration in developing hydrogels for applications in bioprinting and tissue engineering. The findings reveal that mechanical properties can vary substantially based on the material sources used, emphasizing the need for careful consideration in hydrogel formulation [118].

## 5. Hydrogel Distinctive Properties Evaluation

**Figure 4:**
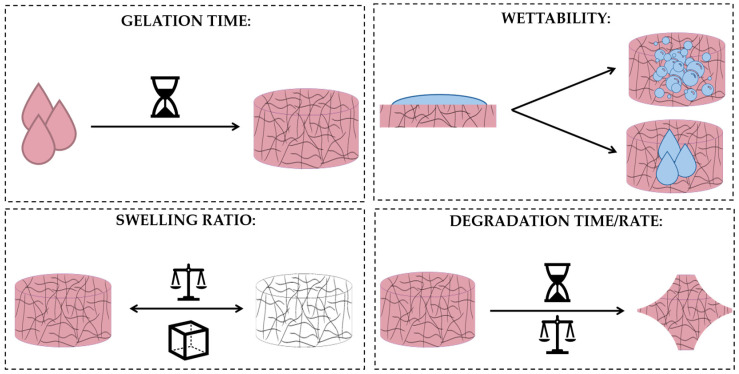
Schematic representation of hydrogel distinctive properties and key parameters to evaluate them. Adapted from Servier Medical Art (https://smart.servier.com), licensed under CC BY 4.0 (https://creativecommons.org/licenses/by/4.0/, accessed on 19 May 2026).

The distinctive properties of hydrogel-based materials include gelation time, swelling ratio, wettability, and degradation time and rate, as visually represented in Figure 4. These characteristics are essential for evaluating the suitability of hydrogels across diverse applications, ensuring optimal performance in both medical and technological contexts. For example, gelation time markedly affects the processing and usage of hydrogels, while the swelling ratio governs their capacity to absorb fluids and deliver drugs effectively. Additionally, wettability is critical for promoting interactions with biological tissues, and the degradation time and rate are crucial for applications that necessitate controlled release or resorption.

### 5.1. Gelling/Gelation Time

Gelation time is defined as the duration necessary for a system to undergo the gelation process, during which a 3D network is established through either chemical or physical cross-linking [119]. This time frame is crucial for determining the properties and potential applications of the resulting gel, as it spans from the initial mixing of components to the appearance of the gel, referred to as the gel point [120]. Consequently, gelation time, or gelling time, refers to the duration required for this transition from the liquid state (sol) to the semi-solid state (gel), essentially marking the time needed to complete the gelation [121].

Gelation time can be qualitatively determined via the visual tube inversion method, also known as the flip-flop method. It consists of the preparation of the hydrogel into a vial or a test tube that must be tilted after a certain amount of time until the hydrogel is stuck at the bottom without liquid leakage [122,123].

Gelation time can be quantitatively assessed through methods such as UV spectroscopy and rheological analysis. UV spectroscopy measures the absorbance of the hydrogel over time to detect changes associated with the stabilization of the hydrogel, marking the conclusion of the gelation process. For instance, a study examined the impact of adding alginate di-aldehyde (ADA) and calcium chloride to a fibrin hydrogel by monitoring absorbance at 350 nm, taking measurements at 30-s intervals for a total of 2 h at 37 °C to maintain physiological conditions. The results indicated that increasing amounts of ADA delayed gelation time, likely due to larger fiber size and density. Conversely, the addition of calcium chloride mitigated the inhibitory effect of ADA on polymerization, resulting in a shorter gelation time [124]. Rheological analysis offers crucial insights into gelation by monitoring the storage modulus (G′) and loss modulus (G″) over time. G′ assesses the material’s ability to store elastic energy, while G″ measures energy lost through viscosity. Gelation occurs when G′ exceeds G″, marking the transition from liquid-like to solid-like behavior. In a related study, researchers examined the effects of hydrogen peroxide concentration and polymer weight percentage on the gelation of enzyme-cross-linked PEG hydrogels. Using a rheometer to maintain temperature and apply constant oscillatory shear, they defined gelation time as the moment G′ equaled G″. Results indicated that higher polymer concentrations and hydrogen peroxide levels led to increased G′. Additionally, with rising polymer and hydrogen peroxide amounts, the water content in the gel decreased, indicating denser polymer networks due to enhanced cross-linking [125]. The tube inversion method provides a macroscopic estimation of the gel point, typically overestimating the gelation time due to the need of the network to reach mechanical strength higher than gravity. To minimize observer bias, this qualitative approach can be formalized using the systematic screening guidelines of ASTM F2900 [126]. Conversely, rheological analysis and UV spectroscopy offer quantitative measurements by capturing the exact onset of viscoelastic elasticity and the real-time kinetics of chemical stabilization, respectively. Therefore, while visual assessments conducted under the ASTM F2900 [126] framework offer a practical, standardized estimate for initial screening, quantitative estimation provides more accurate and reproducible quantitative data. Moreover, dynamic mechanical evaluations following ASTM D4473 [127] standard can support reproducible rheological analysis.

### 5.2. Swelling Ratio

The swelling ratio of a hydrogel is defined as the fractional increase in its weight due to water absorption, indicating its capacity to retain moisture [128]. Within this context, the sol fraction refers to the portion of the polymer that is not part of the cross-linked network, whereas the gel fraction represents the polymer involved in cross-linking [128]. The swelling ratio can be evaluated as the difference between the weight of fully swollen gel and the weight of dry gel divided by the weight of dry gel [129], a gravimetric measurement reported within the ASTM F2900 [126] standard.

Given their extensive applications in areas such as implants and contact lenses, the volume of the swollen hydrogel is often more critical than just the mass increase. A common method for evaluating volume expansion involves measuring the hydrogel’s dimensions before and after swelling, followed by calculating the volume swelling ratio based on the known densities of both the polymer and the solvent [2] or by calculating the density of the hydrogel [130].

Numerous studies have underscored the significance of swelling capacity in hydrogels and the necessity for accurate measurement of this parameter. Griveau et al. explored the interactions between DGL/PEG hydrogels and an effervescent pair composed of glacial acetic acid (Gaa) and potassium carbonate (Pc). They discovered that all hydrogel compositions prepared with the Gaa:Pc pair exhibited an increased swelling ratio, suggesting that the effervescent reaction and resultant pH changes enhanced the hydrogels’ water-absorption capabilities [131]. Similarly, Saeedi et al. investigated the swelling behavior of chitosan-based hydrogels, finding that both the pH of the surrounding medium and the cross-linking density significantly influenced swelling. They observed that lower pH levels enhanced swelling due to chitosan’s cationic nature, while higher cross-linking densities led to reduced swelling ratios due to the creation of a more compact network structure [132].

### 5.3. Wettability

Wettability describes a liquid’s capacity to maintain contact with a solid surface, influenced by the balance between adhesive forces—the attraction between the liquid and the solid—and cohesive forces—the attraction among the liquid molecules [133]. When adhesive forces prevail, a liquid spreads on a surface, indicating high wettability. In contrast, stronger cohesive forces cause the liquid to form droplets, signifying low wettability. This interaction is commonly evaluated through contact angle measurements that can be contextualized within the standardized criteria of ASTM D7334 [134], a framework mapping the junction of the liquid, solid, and vapor, aiding in the robustness of the final results. A smaller contact angle demonstrates better wettability—hydrophilic surfaces—while a larger angle indicates poorer wettability—hydrophobic surfaces [133].

Contact angle analysis can be utilized to investigate how the incorporation of different components alters the surface properties of hydrogels. For instance, one study demonstrated that adding graphene oxide to a calcium alginate hydrogel increased its surface hydrophobicity compared to the pure calcium alginate hydrogel [135]. Additionally, contact angle measurements from another investigation showed that phenolic groups integrated into a HA hydrogel imparted super-hydrophilic characteristics, while the inclusion of phenolic groups in a gelatin hydrogel enhanced its hydrophobicity [136].

Additionally, contact angle measurements can be exploited to evaluate the water absorption kinetics of hydrogels. Indeed, a recent study utilized contact angle analysis to determine the water droplet absorption time, directly quantifying the period required for the liquid to be fully absorbed by the hydrogel matrix [137].

### 5.4. Degradation Time and Degradation Rate

Degradation time can be defined as the duration required for a hydrogel to completely degrade its structure, while degradation rate refers to the overall mass loss from the initial hydrogel over time [138]. The degradation time and degradation rate experiments can be done in accordance with the ASTM F1635 standard to minimize experimental discrepancies and collect more accurate and reliable results [139].

In a study by Asadi et al., the degradation rate was evaluated as mass loss over time for pure GelMA, GelMA-containing eggshell membrane (GelMA/EMF), and GelMA containing both eggshell membrane and propolis (GelMA/EMF/P) [140]. The results highlighted that the addition of both materials into GM decreased the amount of mass loss over time, with pure GelMA exhibiting a mass loss of 90% in 15 days, while GelMA/EMF and GelMA/EMF/P showed a reduced mass loss of 83% in the same period. From a mechanistic standpoint, this occurs because the incorporation of secondary components establishes strong interactions with the primary polymeric chains, restricting the mobility of amorphous segments and reducing both swelling and porosity. Generally, in hydrogel systems, the addition of specific components can lead to a significant variation in the degradation rate driven by these newly formed interactions within the network. Conversely, chemical modifications due to the incorporation of oxidized components can result in an accelerated degradation rate. For instance, in a study involving HA tyramine hydrogels, the introduction of oxidized segments caused a decrease in the degradation time compared to hydrogels without oxidized segments [141]. Mechanistically, a higher degree of oxidation can break the primary polymeric backbone into short-chain fragments. This severe structural fragmentation reduces network packing, increases susceptibility to hydrolytic cleavage, and prevents the formation of a cohesive matrix, driving an ultra-fast mass loss and matrix dissolution.

## 6. Cytocompatibility Evaluation

**Figure 5:**
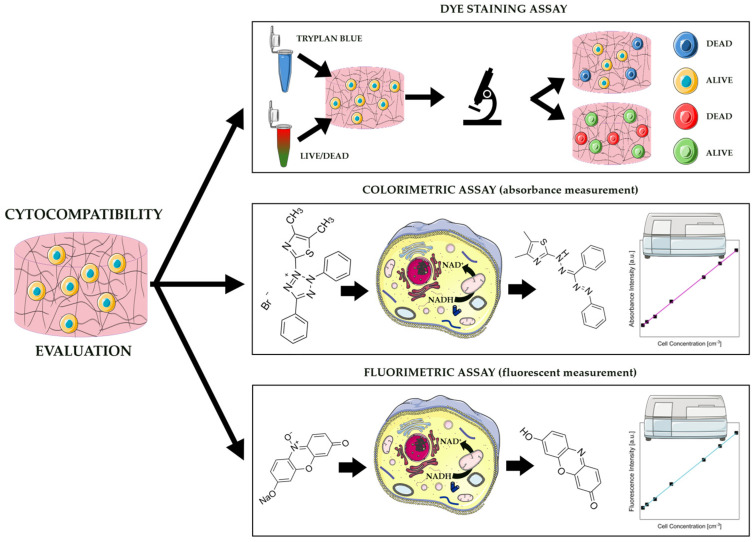
Schematic representation of the working principle of different assays commonly used to evaluate the cytocompatibility of hydrogels. Adapted from Servier Medical Art (https://smart.servier.com), licensed under CC BY 4.0 (https://creativecommons.org/licenses/by/4.0/, accessed on 19 May 2026).

Cytocompatibility evaluation is essential for assessing the health and viability of cells in various experimental contexts, particularly in the development of pharmaceuticals and biomaterials. This evaluation involves measuring the number of healthy/proliferating cells in a sample, which is crucial for understanding the effects of drugs or chemical agents on cellular responses. In this context, the ASTM F2900 [126] standard provides critical recommendations to successfully manage cell viability protocols within 3D embedded scaffold structures to support data reproducibility. Since a standardized analytical assay is not explicitly defined within this framework, various assays are employed to determine cell viability, including dye staining assays, colorimetric assays, and fluorometric assays. The working principles of the assays are reported in Figure 5. It is crucial to carefully consider factors such as the limit of detection, sensitivity, and information needed when selecting an appropriate assay, as these elements directly impact the reliability of the results. A comprehensive cytocompatibility evaluation is essential for ensuring the safety and efficacy of therapeutic agents and materials in biological applications [142].

### 6.1. Dye Staining Assays: Trypan Blue and Live/Dead

Trypan blue (3,3′-[(3,3′-dimethyl(1,1′-biphenyl)-4,4′-diyl)bis(azo)]bis(5-amino-4-hydroxy-2,7-naphthalenedisulfonic acid) tetrasodium salt) is a blue dye commonly used to assess cell viability based on the dye exclusion principle: live cells, with intact membranes, exclude the dye and remain colorless, whereas dead cells (necrosis or late apoptosis), with compromised membranes, take up the dye and appear blue [143]. Trypan blue has been employed in several studies focused on 3D cell cultures. For example, one study investigated L929 fibroblast proliferation on genipin cross-linked gels, calculating the percentage of cell proliferation by comparing the number of viable cells after 48 h to the initial seeding density. The findings revealed a significant increase in cell numbers on the genipin cross-linked gels, indicating that these gels effectively supported fibroblast proliferation [144]. In another study, trypan blue staining was utilized to evaluate the viability of lymphocytes and U937 cells in the presence of various hydrogel concentrations and incubation times. This analysis allowed researchers to assess cell death levels and confirmed that both lymphocytes and U937 cells remained viable and metabolically active under specific experimental conditions [145].

Live/dead assays facilitate the simultaneous detection of live and dead cells using two fluorescent sensors that emit at different wavelengths, such as green/red or red/blue dual fluorescence. These assays utilize inclusion dyes, which require both functional intracellular enzymes and intact cell membranes to accurately measure cell viability and metabolic activity. The mechanism involves the cleavage of non-fluorescent molecules by cytoplasmic esterases, leading to the formation of detectable fluorescent compounds [146]. In contrast, exclusion dyes are cell-impermeable DNA-binding dyes that can penetrate only cells with compromised membranes, emitting distinct fluorescence that indicates non-viable cells [142,146]. Examples of inclusion dyes are calcein, carboxyfluorescein, and fluorescein diacetate, while exclusion dyes are propidium iodide and 7-aminoactinomycin D [146].

These live/dead assays have found extensive application in biomaterials research. For instance, Nuttelman et al. employed the assay to evaluate the viability of human MSC seeded onto various hydrogel surfaces. The results provided visual confirmation of cell attachment and spreading, particularly showing enhanced adhesion and survival on pre-mineralized Poly(ethylene glycol) diacrylate hydrogels compared to non-mineralized surfaces, thus supporting the hypothesis that mineralization enhances cell viability [147]. Lan et al. utilized the live/dead assay to assess the viability of HepG2 cells encapsulated within alginate hydrogels over a two-week culture period. The results demonstrated that, despite limited proliferation due to the lack of adhesion to the alginate matrix, the encapsulated cells remained viable [148].

### 6.2. Colorimetric Assay: MTT and Enhanced MTT Assay

#### 6.2.1. MTT Assay

The MTT reagent (3-(4,5-dimethylthiazol-2-yl)-2,5-diphenyl-2H-tetrazolium bromide) is a mono-tetrazolium salt with a positively charged quaternary tetrazole ring and three aromatic rings. It is a violet-blue water-insoluble compound that can be reduced by metabolically active cells into formazan. Indeed, viable cells reduce MTT to formazan through enzymatic activity, primarily involving oxidoreductase and dehydrogenase enzymes [149]. The MTT assay has been widely used for nearly four decades to evaluate cell proliferation, viability, drug cytotoxicity, and mitochondrial activity. However, its application has extended to infer secondary processes, such as cell viability, which can lead to misinterpretation of results. Indeed, the reduction of MTT primarily indicates metabolic activity rather than direct cell viability [150]. Moreover, the dissolution of formazan crystals required the addition of dimethyl sulfoxide (DMSO), acidified isopropanol, or a solution of sodium dodecyl sulfate (SDS) in diluted hydrochloric acid directly in the cell-seeded plate, so MTT assay is considered an endpoint assay [149,150]. The application of the MTT assay to 3D hydrogels is severely limited by mass transport barriers that impede uniform reagent diffusion [151] and prevent the complete extraction of insoluble formazan crystals from the dense polymer matrix. This issue is further compounded by biochemical interferences, such as non-specific binding of the dye to matrix components, and optical obstacles like gel opacity and meniscus distortion that disrupt absorbance measurements [150]. Additionally, the development of an internal hypoxic gradient induces metabolic heterogeneity, causing the assay to artificially underestimate cell viability by mischaracterizing healthy but quiescent cells in the gel core as dead [152].

Some examples of MTT assay applications in hydrogel-based studies include the assessment of Islam et al. to test the in vitro cytotoxicity of a chitosan scaffold, revealing that the cationic nature of chitosan enhances interaction with negatively charged cell membranes, promoting cell adhesion and proliferation. Additionally, the study found that chitosan scaffolds do not release toxic products to human fibroblast cells [153]. Similarly, Capella et al. investigated the cytotoxicity of poly-NIPAM) hydrogel using MTT assays on different cancer cell lines. The biocompatibility of poly-NIPAM was assessed through the comparison of cell viability on hydrogel surfaces and on 2D without exposure to hydrogels [154].

#### 6.2.2. Enhanced MTT Assay (MTS, XTT, and WST Assays)

Enhanced tetrazolium reagents represent an advancement over the traditional tetrazolium assays, streamlining the process of measuring cellular metabolic activity. These reagents simplify the assay protocol by eliminating the need for a separate solubilization step to dissolve the formazan precipitate, which is typically formed during the reduction of tetrazolium salts by metabolically active cells [142]. Enhanced tetrazolium reagents are used in combination with intermediate electron acceptor reagents, such as PMS (phenazine methyl sulfate) and PES (phenazine ethyl sulfate). These acceptors penetrate viable cells, undergo a reduction in the cytoplasm or at the cell surface, and subsequently exit the cells, where they facilitate the conversion of tetrazolium into a soluble formazan product [155]. By enabling the direct formation of a soluble formazan product, these reagents enhance the efficiency and applicability of tetrazolium-based assays in various biological studies [142].

The MTS reagent (3-(4,5-dimethylthiazol-2-yl)-5-(3-carboxymethoxyphenyl)-2-(4-sulfophenyl)-2H-tetrazolium) is a negatively charged compound that requires an intermediate electron acceptor to facilitate the reduction of the tetrazolium by mitochondrial dehydrogenase enzyme, leading to the formation of a colored formazan product [156,157].

The MTS assay is widely used to evaluate cell viability and proliferation in response to different treatments. For example, it has been employed to assess how various treatments influence chondrocytes viability in the presence of the pro-inflammatory cytokine IL-1β, providing insights into their protective effects against inflammation-induced cell damage [158]. Additionally, the MTS assay has been applied to investigate the impact of functionalized hydrogels on cell proliferation. Results indicated that RADA16-FPG hydrogel significantly inhibits fibroblast proliferation and moderately affects keratinocyte proliferation, while trends in reduced proliferation are also observed in the RGD scaffolds [159].

The XTT reagent (2,3-Bis-(2-Methoxy-4-Nitro-5-Sulfophenyl)-2H-Tetrazolium-5-Carboxanilide) can be extracellularly reduced by NADH produced in the mitochondria via trans-plasma membrane electron transport and an electron mediator, leading to the formation of formazan [160].

The XTT assay is commonly used to assess the density and viability of cells adhered to various types of microcarriers (MHs). Rauer et al. conducted a study to assess the cytocompatibility of poly(3,4-ethylenedioxythiophene):polystyrene sulfonate MHs, employing the XTT assay with L929 mouse fibroblast cells [161]. The results indicated high viability rates for cells cultured on both low-crystalline and high-crystalline MHs by day 9 of culture. These findings confirm the non-cytotoxic nature of the MHs, supporting their suitability for cell culture applications. Additionally, the XTT assay has been used to compare the cytocompatibility of hydrogels with and without the addition of conjugates. In particular, Wang et al. compared the cytocompatibility of antibiotic-conjugated hydrogels and hydrogels without antibiotics. The results indicate that both hydrogel formulations exhibited no cytotoxic effects on the NIH3T3 mouse fibroblast cell line [162].

WST (water-soluble tetrazolium salts) are a class of reagents that have been developed for use in colorimetric assays to measure cellular metabolic activity, particularly through the detection of NADP(H) [163]. WST compounds generate a highly water-soluble formazan product via mitochondrial dehydrogenase enzymes in the presence of an intermediate electron acceptor, such as 1-methoxy PMS [142]. Several WST derivatives, including WST-1, WST-3, WST-4, WST-5, WST-8, and WST-9, have been synthesized and are widely utilized in various biological applications [163]. Among them, the WST-1 and WST-8 assays are commonly used to quantify the metabolic activity of cells, which serves as an indicator of cell viability and helps assess the potential toxic effects of hydrogels. A study reported that, after cell treatment with gel-only and gel-PLL (ε-poly-l-lysine), metabolic activity remained unchanged on day 1, but increased on day 2 and 3, supporting the biocompatibility of the hydrogels for potential wound dressing applications [164]. Additionally, Patel et al. used the WST-8 assay to demonstrate the biocompatibility of the hydrogels by confirming that they did not negatively impact the viability of human dermal fibroblast cells [165].

### 6.3. Fluorometric Assay: Alamar Blue

The Alamar Blue reagent (7-Hydroxy-3H-phenoxazin-3-one 10-oxide), also known as resazurin, is a weakly fluorescent, non-toxic, and cell-permeable phenoxazine dye. Since Alamar Blue is cell-permeable, it is internalized by cells and metabolically reduced in the cytoplasm of living cells by cytosolic, microsomal, and mitochondrial dehydrogenase or reductase enzymes, producing resorufin (7-Hydroxy-3H-phenoxazin-3-one), a highly fluorescent pink molecule [166]. The Alamar blue assay is widely used in preclinical pharmaceutical research to evaluate drug candidates’ safety and efficacy. It helps screen potential drugs for cytotoxicity, allowing researchers to identify compounds with adverse effects and prioritize safer candidates for further development. Moreover, the assay can be employed to study the effects of growth factors or cytokines on cell proliferation, providing insights into cellular responses to various stimuli [166].

Several studies have demonstrated the potential of Alamar Blue in three-dimensional (3D) hydrogel systems: for instance, a study on A549 cells encapsulated in a fibrin gel scaffold found that residual resazurin/resorufin remained trapped in the fibrin gel, interfering with subsequent measurements, while prolonged exposure to resazurin was associated with reduced cell numbers and altered cell morphology, suggesting possible cytotoxicity over time. Therefore, the study recommends limiting resazurin incubations to no more than two sequential time-lapse measurements in a 3D matrix to mitigate the risk of resazurin-associated cytotoxicity and ensure accurate viability assessments [167].

Another study evaluated cell metabolic activity in alginate-based hydrogels, revealing that plain alginate inhibited cell growth, while the addition of fibronectin significantly improved cell viability [168]. Additionally, Alamar Blue was used to assess chondrocyte growth in chitosan-gelatin and chitosan-gelatin-sodium alginate scaffolds, demonstrating that both scaffolds support chondrocyte proliferation; however, the chitosan-gelatin scaffold proves more effective in promoting cell proliferation and maintaining the appropriate chondrocyte phenotype, making it particularly suitable for cartilage tissue engineering [169].

## 7. Application-Based Hydrogel Properties Evaluation

**Figure 6:**
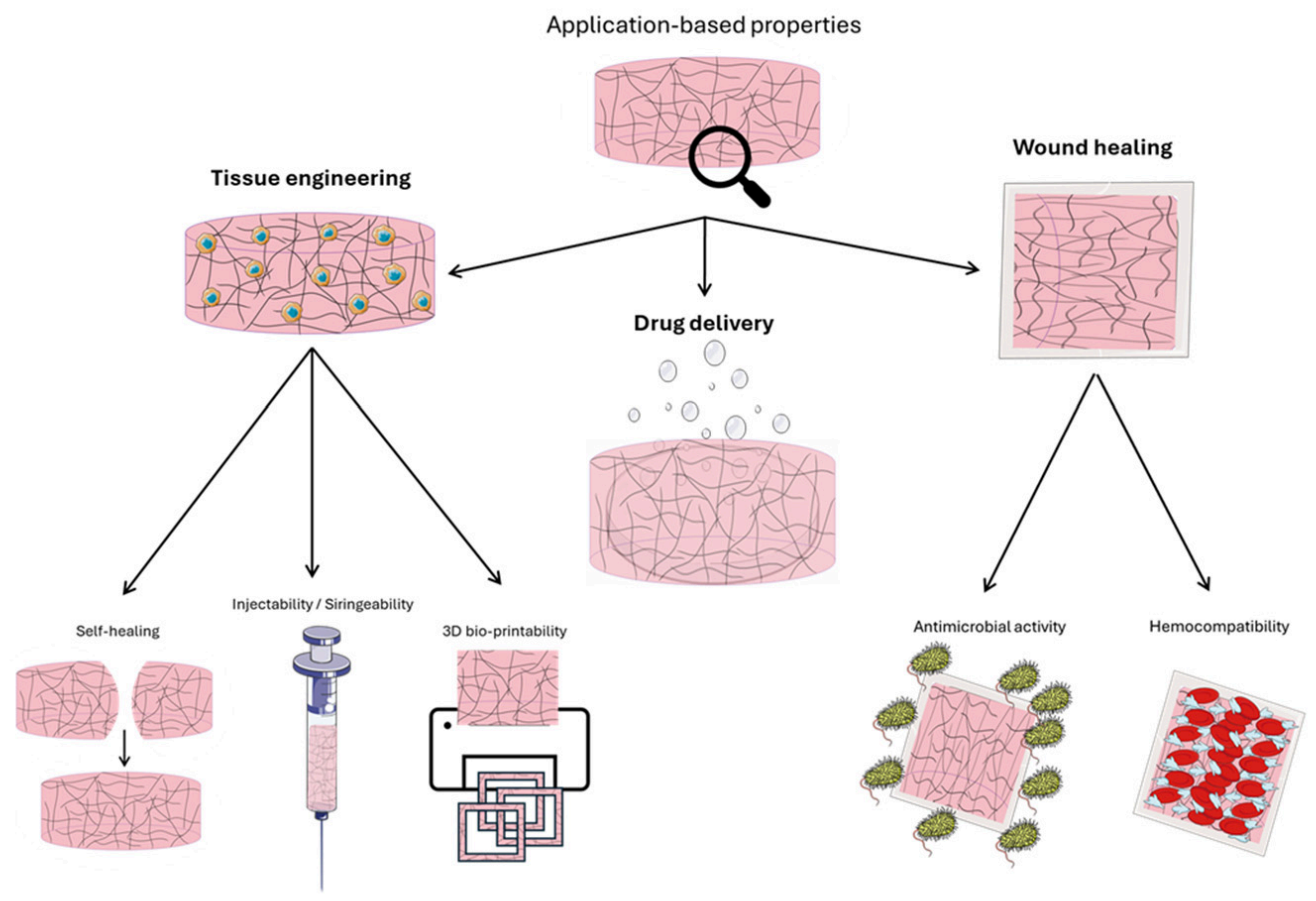
Schematic representation of the methods commonly used to test the suitability of hydrogels for specific applications. Adapted from Servier Medical Art (https://smart.servier.com), licensed under CC BY 4.0 (https://creativecommons.org/licenses/by/4.0/, accessed on 19 May 2026).

Hydrogels emerged as candidate materials for multi-purpose applications within the biomedical field, Figure 6 illustrates some of the possible application of hydrogel within this field. Particularly, their high-water content and tissue-like mechanical properties guarantee the creation of an environment able to support cell growth and interactions. Hydrogels can be used as a scaffolding platform for cells to create biomimetic environments that closely resemble the extracellular matrix, facilitating investigations into developmental processes and enhancing tissue engineering efforts. This capability deepens the understanding of cellular behavior and holds significant promise for a range of biomedical applications, including cancer research, where hydrogels can simulate disease progression and aid in the screening of anti-cancer therapies [170].

Beyond their use as scaffold in 3D cell culture, some hydrogels exhibit remarkable self-healing properties similar to those found in human tissues. This self-healing capability is crucial for applications that require materials to endure mechanical stress. Researchers have investigated both covalent and non-covalent interactions to improve the mechanical properties of hydrogels, enabling these materials to autonomously heal from damage. Such advancements open new avenues for employing hydrogels in dynamic biomedical applications, including regenerative medicine and tissue engineering [171].

Hydrogels are also gaining attention for their potential in drug delivery systems. Their porous structure allows for effective drug loading while safeguarding therapeutic agents against harsh environments. By adjusting the cross-linking density of the hydrogel matrix, researchers can finely tune the drug release rate, making hydrogels versatile carriers for various pharmaceutical compounds [172]. Moreover, their adaptability extends to biomonitoring and the development of smart drug delivery systems [173,174]. However, to ensure that each hydrogel formulation is optimized for its intended use, rigorous testing of its performance is essential. Characterization strategies are critical for various applications, including modeling disease progression, drug release, tissue engineering, regenerative medicine, 3D bioprinting, and wound dressing. Understanding how hydrogels perform in specific contexts will be explored in detail in the following sections. By systematically assessing these characteristics, researchers can refine and enhance the properties of hydrogels, ultimately leading to better patient outcomes and more effective treatment strategies. This comprehensive approach not only facilitates the creation of tailored hydrogel formulations but also supports the advancement of innovative therapies across a broad spectrum of medical applications.

### 7.1. Drug Delivery Systems

Drug delivery refers to the techniques and procedures used to administer pharmaceutical compounds to achieve therapeutic effects in humans or animals. This field involves the development of innovative materials and carrier systems that enhance drug delivery, considering that these systems are designed to transport drugs to their intended site of action in a controlled manner, ensuring timely and appropriate release of the drug [175].

Key characteristics of effective drug delivery systems include the ability to control release rates, target specific tissues or cells, and maintain stability during storage and administration. Hydrogels facilitate prolonged and sustained release of drugs over extended periods, regulated through various strategies—including adjusting the mesh size of the hydrogel to control drug diffusion rates, network degradation via hydrolysis or external triggers, incorporating chemical bonds between drugs and polymer chains for sustained release through strong interactions, utilizing electrostatic interactions within the hydrogels to enable the simultaneous delivery of multiple drugs, and including hydrophobic components to ensure effective release of hydrophobic drugs [28].

Hydrogel drug release capabilities can be evaluated by loading a known quantity of dissolved drugs into the hydrogel matrix and analyzing the release profile. The process involves preparing the hydrogel, incorporating the drug, and covering its surface with a release medium to facilitate drug diffusion. Samples of the surrounding liquid are collected at set intervals to monitor drug release, with concentrations quantified using a spectrophotometer based on absorbance measurements at specific wavelengths. This quantification relies on Lambert-Beer law or a calibration curve constructed from known drug concentrations. By tracking absorbance over time, researchers can calculate cumulative release and gain insights into the drug release kinetics and performance of the hydrogel as a drug delivery system [176,177].

For instance, a study by Liu et al. examined the drug release properties of silk fibroin hydrogels containing FITC-labeled BSA as a model protein drug. The researchers used a calibration curve to quantify drug release over time, revealing a rapid burst release in the first 12 h, followed by sustained release up to 72 h, with cumulative release values reaching 168 h [176]. Similarly, Suhail et al. investigated the drug release capabilities of β-Cyclodextrin-Based Smart Hydrogels, employing the Lambert-Beer law to determine released drug concentration, enabling the calculation of cumulative theophylline release. Their findings demonstrated significant in vitro release of theophylline over 36 h, with swelling observed for up to 72 h, indicating a prolonged drug release profile [177]. Moreover, Baishya et al. estimated the drug release kinetics through the application of mathematical models [178].

### 7.2. Tissue Engineering

Tissue engineering (TE) is dedicated to creating biological substitutes that can restore or enhance the function of damaged tissues and organs, drawing on principles from engineering, biology, materials science, and medicine. The growing need for TE underscores the limitations of traditional treatments for organ failure, especially given the insufficient supply of organ donors [179].

Hydrogels have become promising biomaterials for TE due to their distinctive properties. For effective application, they must fulfill specific requirements: injectability for minimally invasive administration, which allows for accurate placement at injury sites and supports personalized medicine; self-healing abilities that enable them to regain structural integrity after damage, essential for long-term use; and printability to create complex 3D structures that replicate natural tissue, facilitating the development of scaffolds that provide necessary mechanical and topographical cues for cellular behavior [179,180,181].

Researchers evaluate hydrogels for their injectability, self-healing, and printability to determine their suitability for TE applications. These evaluations offer crucial insights into hydrogel performance and guide the selection of appropriate materials for regenerative medicine, ultimately advancing the development of effective therapeutic solutions.

#### 7.2.1. Injectability and Syringeability

Syringeability and injectability are critical parameters for ensuring effective hydrogel delivery in medical applications. Syringeability refers to the ease of expelling a hydrogel through a needle, while injectability evaluates the performance of the syringe during subcutaneous injection by measuring the force required for administration [182]. Currently, there are no standardized international methods to test these parameters, though various experimental procedures are documented in the literature [182].

Syringeability can be quantified as the percentage of hydrogel expelled from the syringe, calculated by applying a constant force for a set duration and measuring the residual hydrogel mass left in the syringe [183]. Alternatively, syringeability can be assessed using specialized instruments designed to measure the ease of hydrogel expulsion, as demonstrated by E. Chiesa et al., who tested hydrogel samples at a constant pressure and measured flow rates suitable for manual injection [184].

On the other hand, injectability can be qualitatively assessed for polymeric solutions, as done in a study where ten participants from different backgrounds were asked to rate how easy it was to draw the solution into the syringe and inject it, using a score from 1 to 4 [185]. Injectability can be quantitatively assessed by measuring the force required to inject the hydrogel during a compression test using a universal testing machine that monitors force versus displacement at a constant crosshead speed [184]. The initial glide force and the maximum force recorded during the test are quantified, with both values needing to remain below 30 N to meet the acceptable limit for subcutaneous injections as defined by Burckbuchler et al. [186].

Note that the absence of standardized international methods for assessing these parameters poses a significant limitation. The varied techniques employed in the literature hinder comparisons and consistency in clinical applications, compromising the optimization of hydrogel formulations and potentially affecting the safety and efficacy of administration procedures. This underscores the urgent need for uniform protocols to evaluate these fundamental aspects.

#### 7.2.2. Self-Healing Capability

Self-healing materials can partially or fully restore their original properties after sustaining damage from external factors, such as mechanical or thermal stimuli, without requiring human intervention. This remarkable capability is particularly prominent in hydrogels, which mimic biological systems through a self-healing mechanism consisting of five key steps: (i) surface rearrangement, (ii) surface approach, (iii) wetting, (iv) diffusion, and (v) randomization. These processes are driven by molecular interactions between fractured surfaces, relying on dynamic and reversible bonds to facilitate regeneration [187,188].

The self-healing abilities of hydrogels can be quantitatively assessed by comparing the mechanical properties of the original and healed hydrogel. For example, a study on a graphene oxide-based crosslinked polyacrylic acid hydrogel demonstrated that the healed sample retained impressive tensile strength and moderate stretchability, achieving nearly 60% stress healing efficiency after 40 h of contact time [189].

Another investigation focused on the self-healing properties of waterborne polyurethane/collagen coatings. In this study, specimens were cut, and the fracture surfaces were hydrated with water vapor before being reassembled for 12 h. The results showed significant self-healing capabilities, with increased collagen content from 0% to 5% enhancing healing efficiency for strain and tensile stress, from 10% and 36% to 76% and 67%, respectively [190].

#### 7.2.3. 3D Bio-Printability

3D bio-printing is a rapidly evolving area in biomedical engineering that employs additive manufacturing techniques to create intricate biological structures. Hydrogels or hydrogel precursors serve as “inks” in this process; when loaded with cells, they are termed bio-inks [191], and when containing bacteria, they are referred to as microbial inks [192]. The printability of hydrogels is essential for their successful application in 3D printing and encompasses several critical parameters, including viscosity, gelation mechanisms, thermal properties, bulk density, and wettability. The 3D Hydrogel-Focused Extrusion (HFE) printing process involves layer-by-layer dispensing of semi-solid polymeric solutions through a movable nozzle. Several factors influence the selection of a polymer for 3D HFE printing, including biocompatibility, mechanical properties, and printability [193]. Achieving an equilibrium between printability and biocompatibility is crucial, as it ensures structural integrity while maximizing cell viability [194]. This equilibrium is often referred to as the “bio-fabrication window”. Within this window, while increased hydrogel viscosity can enhance the quality of the scaffold, it may also pose risks to cell membranes, thereby necessitating a careful compromise [195].

A primary challenge in using HFE-based 3D printing techniques is ensuring print quality, as hydrogels are largely composed of water, which can hinder the production of precise and reproducible structures. Print quality includes several aspects: printability, extrudability, printing accuracy, and printing fidelity [194]. Printability refers to the ability of an ink to achieve extrusion while maintaining shape fidelity [196], extrudability denotes the consistent extrusion of the hydrogel through the nozzle [197], printing accuracy measures how closely the printed construct matches the intended size and spatial location defined by the original CAD model [198], and printing fidelity assesses how well the printed construct adheres to the original design file in terms of geometry [199].

The development of suitable inks for 3D HFE printing systems is currently in its early stages of research. However, several studies focusing on hydrogel printability have been published. For instance, one study investigated composite hydrogels made from collagen and chitosan, aiming to identify optimal concentration ratios that facilitate the formation of continuous filaments while minimizing challenges such as hydrogel droplet formation and needle clogging. This research is pivotal for refining the printing process and enhancing the reliability of the printed constructs [200].

Another study examined the printing accuracy and fidelity of hydrogel solutions, specifically assessing their ability to maintain desired shapes and dimensions after the printing and cross-linking processes [201]. In this study, the bioprinter was programmed to create an arbitrary surface, and the quality of each printed gel was evaluated based on its adherence to the specified dimensions. Printing accuracy was quantified by calculating the ratio of the target area to the actual printed area, resulting in a percentage that reflects how closely the printed gel aligned with the intended design.

### 7.3. Wound Healing

Wound healing is a multifaceted process that begins with tissue injury and involves various cell types, cytokines, and the vascular system. It progresses through distinct phases. The initial response includes vasoconstriction and platelet aggregation to achieve hemostasis. This is followed by an inflammatory phase, during which neutrophils and immune cells release mediators that facilitate angiogenesis, thrombosis, and re-epithelialization, while fibroblasts deposit extracellular matrix components. The proliferative phase sees the formation of granulation tissue, cell migration, collagen synthesis, and neovascularization. Finally, the maturation phase ensures tissue remodeling and wound contraction, although the regenerated tissue typically regains only about 80% of its original strength [202,203,204]. In some cases, the human body may be incapable of healing certain wounds autonomously, necessitating the application of wound dressings to facilitate the healing process. A wound dressing is defined as any material used in direct contact with a wound to promote healing, absorb exudates and fluids, and reduce the risk of infection. The primary role of wound dressings is to support the local wound environment, thereby enhancing effective wound healing [205].

Among the various types of wound dressing materials, hydrogels have proven to be particularly effective in wound care. For hydrogels to function efficiently as wound dressings, they must act as a barrier against external microbial contaminants while also exhibiting broad-spectrum antibacterial activity. Another critical factor in selecting wound dressing materials is hemocompatibility, especially for burn dressings that frequently interface with various body fluids (e.g., in second- and third-degree injuries). A dressing material lacking hemocompatible properties can trigger an adverse immune response in the patient, leading to issues such as thrombosis formation, inflammation, and foreign body reactions. Such responses can ultimately hinder the healing process [206].

#### 7.3.1. Antimicrobial Activity

The antibacterial and antiviral properties of a compound can effectively kill bacteria and viruses or inhibit their growth without significantly harming nearby tissues [207]. One common technique for assessing antibacterial activity is the agar well-diffusion method, where a hydrogel sample is placed in a well punched into an agar plate inoculated with target bacteria. The effectiveness is determined by measuring the diameter of the inhibition zone around the well, reflecting the extent of bacterial growth inhibition. For example, a study tested the antimicrobial activity of a hydrogel made from quaternary ammonium chitosan (HACC), PVA, and polyethylene oxide (PEO) against *S. aureus* and *E. coli*. The results revealed significant antibacterial effects due to the incorporation of HACC, which interacts with the negatively charged surfaces of bacterial cells, causing structural alterations and the release of cellular components, ultimately leading to bacterial death [208].

Another method for evaluating antibacterial activity is the optical density (O.D.) method, following the ASTM E2149-01 standard [209]. In this approach, the hydrogel is immersed in a medium containing the target bacteria, and the O.D. of the medium is measured at 650 nm to assess bacterial proliferation. For instance, Tsao et al. studied the antimicrobial activity of chitosan–γ-poly(glutamic acid) polyelectrolyte complex hydrogels against *S. aureus* and *E. coli*, finding enhanced antibacterial properties influenced by the hydrophilicity of the materials, which helped prevent microbial adhesion [210].

#### 7.3.2. Hemocompatibility Assessment

Evaluating hemocompatibility is essential to ensure that wound dressing materials can safely interact with blood and body fluids without causing harmful immune responses. It is defined as the measurement of either the thrombotic response to materials in contact with blood or the damage to red blood cells from exposure to those materials. Researchers employ various testing methods to assess hemocompatibility, guiding the development of effective and safe wound care solutions [211].

Several assays are significant for evaluating hemocompatibility, including the hemolysis assay, coagulation assay, prothrombin time (PT) assay, partial thromboplastin time (PTT) assay, and complement activation test [212]. For instance, one study investigated the hemocompatibility of xyloglucan-PVA based hydrogel (XG-PVA) films through PT and PTT tests, finding that the XG-PVA film did not induce platelet activation or thrombus formation, with all measured parameters remaining within the normal human range [213]. In another study, the hemocompatibility of PVA-sodium alginate xerogel membranes was assessed by quantitatively measuring the hemoglobin release into the supernatant spectrophotometrically [214]. The hemolysis values for these membranes ranged from 0.06% to 1.22% as the sodium alginate content increased, categorizing them as non-hemolytic materials (hemolysis < 2%) according to ASTM classification [215].

## 8. Conclusions

Hydrogels represent a remarkable class of materials with unique properties that make them invaluable in various applications, especially in the biomedical field. Their three-dimensional, hydrophilic polymer networks allow for significant water absorption and retention, closely mimicking natural tissue environments and supporting diverse biological functions. Although significant progress has been made in understanding their classification, preparation, and applications, there remains a critical need for comprehensive studies focused on their characterization.

Characterization is vital to elucidating the physicochemical, morphological, and mechanical properties of hydrogels, directly influencing their performance across different applications. The techniques discussed in this review provide valuable insights into the structure and behavior of hydrogels, enabling researchers to tailor their properties for specific uses. Understanding biocompatibility and application-specific characteristics is essential for their successful integration into medical devices, drug delivery systems, and tissue engineering scaffolds.

This review aimed to address the gap in the literature by organizing key aspects of hydrogel characterization into distinct sections, detailing relevant techniques, their purposes, advantages, and limitations. By doing so, it not only highlighted the importance of each technique in understanding hydrogels but also assisted researchers in determining the most suitable applications for these materials. While existing ISO and ASTM standards provide a foundational framework for hydrogel characterization, significant technical gaps remain. Addressing these requires a collaborative effort among experts to develop specialized guidelines that account for the unique, complex behavior of hydrogel-based materials. Notably, for the most frequently investigated morphological and mechanical properties, the absence of unified standard operating procedures creates a disjointed research environment. Consequently, current findings often lack comparability and reproducibility, highlighting a critical need for collaborative development of standardized guidelines tailored to the unique behavior of hydrogel-based systems.

As hydrogel research continues to evolve, interdisciplinary collaboration will be crucial in unlocking new possibilities. By integrating insights from materials science, biology, and engineering, researchers can develop innovative hydrogel formulations that address current challenges in healthcare and other industries. Specifically, future work must focus on the standardization of experimental techniques for testing application-based properties, such as injectability and syringeability. Furthermore, newly developed formulations should be characterized according to their specific intended applications. To support this systematic approach, Table 1 presents a methodological roadmap for hydrogel characterization across key biomedical applications, defining the characterization strategies for each application, and details the rationale behind the selection of each testing technique. Finally, for a systematic overview of the data obtainable through each technique discussed in this review, we refer the reader to Table A1 in Appendix A. The potential of hydrogels to revolutionize areas such as wound healing, drug delivery, and regenerative medicine is immense, and ongoing exploration of their properties will undoubtedly lead to significant advancements.

## Figures and Tables

**Table 1:** Methodological roadmap for hydrogel characterization across key biomedical applications, mapping maturity levels, characterization strategy for each biomedical application including suggested techniques and the rationale for the choices.

Application	Maturity Level	Characterization Strategy	Rationale
Drug delivery system	High (Clinically Viable/ Commercial)	SEM/SAXS	Determines network porosity and mesh size to define maximum compatible drug molecular weight.
		IR	Verifies drug incorporation, distinguishing between physical entrapment and covalent bonding.
		XRD	Confirms drug crystallographic state, tracking amorphous stabilization or crystal growth.
		DSC	Maps thermodynamic profiles to ensure molecular drug dispersion without phase transitions.
		Tensiometer	Maps how component incorporation alters surface wetting energy.
		Rheology	Identifies passive diffusion versus stimuli-induced release, quantifying trigger shear thresholds.
		UV-Vis spectroscopy	Map the time-dependent release profiles and active cargo diffusion kinetics.
		Gravimetric analysis	Tracks long-term polymer degradation time under physiological conditions.
		Cytocompatibility	Evaluates independent cytotoxicity profiles of the raw composite and its degradation byproducts.
Tissue engineering	Medium (Experimental/ Pre-clinical)	IR/NMR	Quantifies cross-linking density and functional group conversion for network stability.
		SEM/TEM	Maps 3D porous micro- and nano-architecture for cell infiltration and matrix interactions.
		UTM/Rheometer	Determines static and dynamic mechanical properties to match target tissue elasticity.
		Rheometer/ UV-Vis spectroscopy	Captures exact gelation time and liquid-to-solid transition parameters.
		Tensiometer	Assesses surface wettability to predict cell–matrix interfacial interactions.
		Gravimetric analysis	Measures swelling ratio to ensure osmotic equilibrium and structural nutrient transport.
			Tracks long-term degradation rate to balance mass loss with new matrix deposition kinetics.
		Self-healing test	Quantifies structural recovery time post-injection to prevent immediate gel migration.
		Injectability/ Syringeability	Determines required extrusion forces for minimally invasive delivery.
		3D Bioprintability	Defines bio-fabrication window and shape fidelity, preventing post-printing structural deformation.
		Cytocompatibility	Evaluate baseline cytocompatibility and cell survival within 3D embedded configurations.
Wound healing patches	High (Clinically Viable/ Commercial)	IR	Verifies binding of active antimicrobial/hydrating components and screens for toxic residual solvents.
		XRD	Confirms predominantly amorphous network structure to guarantee maximum patch flexibility on skin
		DSC	Maps matrix thermal stability to prevent biopolymer denaturation at wound core temperature.
		TGA	Quantifies bound and unbound water content to predict gel drying kinetics under air exposure.
		UTM	Evaluates elongation at break and Young’s modulus to match skin mechanical deformation.
			Quantifies peel and shear adhesive strength on wet biological tissue interfaces.
		Rheology	Determines resilience under compressive bandage loads, preventing liquefaction or structural failure.
			Measures quantitative gelation time for precise in situ curing.
		Gravimetric analysis	Quantifies dynamic water-absorption and swelling ratios to prevent fluid accumulation.
			Optimizes moisture evaporation rates to maintain a balanced, non-drying humid interface.
			Tracks long-term enzymatically simulated degradation kinetics to predict clinical patch wear time.
		Cytocompatibility	Evaluates in vitro cytotoxicity to ensure dermal fibroblast and keratinocyte survival.
		Hemocompatibility	Confirms lack of hemolysis or adverse coagulation loops upon direct contact with bleeding lesions.
		Antibacterial activity	Verifies active antimicrobial and barrier efficiency against common wound pathogens.
3D Models for Cancer & Pharma Research	Low to Medium (Experimental/ In Vitro)	IR	Verifies matrix cross-linking efficiency and screens for toxic unreacted monomers.
		DSC	Maps thermal transition points of temperature-sensitive matrices to ensure rapid, stable gelation at 37 °C.
		AFM—force spectroscopy analysis	Quantifies local microscopic matrix stiffness variations in driving cell mechano-transduction loops.
		Rheology	Measures bulk gel elastic modulus, replicating tumor rigidity to trigger chemo-resistance.
			Captures liquid-to-solid transition kinetics to ensure network formation within cell-survival windows.
		Gravimetric analysis	Measures fluid absorption, ensuring volumetric expansion does not distort 3D geometries.
			Tracks cell-driven enzymatic degradation and network remodeling during tumor invasion.
		Cytocompatibility	Ensures material non-toxicity so drug-screening responses depend solely on therapeutic candidates
		3D Bioprintability Testing	Optimizes bio-fabrication windows, balancing filament shape fidelity with cell shear protection.

## Data Availability

No new data were created or analyzed in this study. Data sharing is not applicable to this article.

## Abbreviations

The following abbreviations are used in this manuscript:

PVA	Poly(vinyl alcohol)
pHEMA	Poly(2-hydroxyethyl methacrylate)
PEG	Poly(ethylene glycol)
IR	Infrared spectroscopy
NMR	Nuclear magnetic resonance
SAXS	Small-angle X-ray scattering analysis
XRD	X-ray diffraction analysis
DSC	Differential scanning calorimetry analysis
TGA	Thermal gravimetric analysis
GXG	Galactoxyloglucan
αCD	α-Cyclodextrin
HA	Hyaluronic acid
NAR	Naringin
^1^H	Hydrogen-1
^13^C	Carbon-13
^17^O	Oxygen-17
GelMA	Gelatin methacryloyl
-NH_2_	Amine groups
SA	Sialic acid
ISO	International standards organization
PNIPAM	GelMA–poly(N-isopropylacrylamide)
NIPAM	N-isopropylacrylamide
CC	Chitosan–catechol
BNNSs	Boron nitride nanosheets
NaCMC-cl-DMAA/AAc	Carboxymethyl cellulose-cross-linked-N’N-dimethyl acrylamide/acrylic acid
Tg	Glass transition temperature
LDC	Lidocaine hydrochloride
BSE	Backscattered electrons
SE	Secondary electrons
ESEM	Environmental SEM
Alg + PPy	Alginate-polypyrrole
Alg/Gel + PPy	Alginate/gelatin-polypyrrole
MSC	Mesenchymal stem cell
FE-SEM	Field emission SEM
UTM	Universal testing machines
G′	Storage modulus
G″	Loss modulus
ADA	Alginate di-aldehyde
Gaa	Glacial acetic acid
Pc	Potassium carbonate
GelMA/EMF	GelMA-containing eggshell membrane
GelMA/EMF/P	GelMA containing both eggshell membrane and propolis
MTT	3-(4,5-dimethylthiazol-2-yl)-2,5-diphenyl-2H-tetrazolium bromide
DMSO	Dimethyl sulfoxide
SDS	Sodium dodecyl sulfate
PMS	Phenazine methyl sulfate
PES	Phenazine ethyl sulfate
MTS	3-(4,5-dimethylthiazol-2-yl)-5-(3-carboxymethoxyphenyl)-2-(4-sulfophenyl)-2H-tetrazolium
XTT	2,3-bis-(2-methoxy-4-nitro-5-sulfophenyl)-2h-tetrazolium-5-carboxanilide
MHs	Microcarriers
WST	Water-soluble tetrazolium salts
gel-PLL	Gel-poly-L-lysine
HFE	Hydrogel-focused extrusion
HACC	Quaternary ammonium chitosan
PEO	Polyethylene oxide
O.D.	Optical density
ASTM	American Society for Testing and Materials
PT	Prothrombin time
PTT	Partial thromboplastin time
XG-PVA	Xyloglucan-PVA based hydrogel

## Appendix A

A comprehensive summary of the information that can be collected from each characterization technique mentioned in the review is reported in Table A1.

**Table A1:** Comprehensive summary of the information that can be collected from each characterization technique mentioned in the review.

Characterization Area	Technique/ Method	Information About	Ref.
Physicochemical properties	IR	Formation and the purity of hydrogel precursors	[40,41]
		2. Successful synthesis of hydrogels	[42,43]
		3. Hydrogel future behavior based on the presence or absence of characteristic peaks	[44,45]
		4. Hydrogel cross-linking	[46]
		5. Interactions among hydrogel precursors	[47,48]
		6. Successful integration of drugs into hydrogels and the potential interactions between the drug and the hydrogel matrix	[49,50]
	NMR	Efficacy of hydrogel and its precursor synthesis	[54,55,56]
		2. Chemical reactions leading to hydrogel formation	[49]
		3. Extent of cross-linking in superabsorbent networks	[57]
		4. Interactions with additives	
		5. Solid-state structural properties and packing configurations	
		6. Polymorphic forms	
		7. Conformational changes that influence gelation properties	
	SAXS	Morphological changes during the gelation process	[54,55]
		2. Hydrogel microstructures	[49,61]
		3. Microstructural changes induced by drug loading into hydrogels	[62]
	XRD	Interactions occurring during hydrogel formation	[47]
		2. Crystallinity of hydrogels	[40]
		3. Degree of microstructure assembly within hydrogels	[66]
		4. Element incorporation into hydrogel matrices	[43]
	DSC	Interactions between the polymers and their cross-linkers	[50]
		2. Drug-hydrogel interactions	[47,48]
		3. Thermo-reversible gelation	[71]
	TGA	Thermal stability of hydrogels	[43,75]
		2. Thermal stability of hydrogel precursors	[40,41]
Morphological properties	SEM	Hydrogel morphological features	[66,85]
		2. Hydrogel internal structure and pore architecture	[86]
		3. Distribution and morphology of particles/molecules within the hydrogel matrix	[87]
	TEM	Hydrogel morphological features	[66,92]
		2. Morphological differences arising from various formation processes	[93]
	AFM	Surface modifications and the effects of chemical agents on materials	[97]
		2. Surface parameters for aiding in the selection of suitable options for various applications	[98]
		3. Topographical features	[99]
Mechanical properties	UTM	Hydrogel mechanical properties	[104]
		2. Impact of varying precursor concentrations on the mechanical properties	[105]
	Rheometer	Hydrogel mechanical properties	[111,112]
		2. Influence of nanoparticle shape on the mechanical properties of hydrogels	[111]
		3. Influence of nanoparticle on mechanical properties and behavior	[112]
	AFM	Elastic properties of hydrogels	[118]
Hydrogel distinctive properties	Visual tube inversion method	Hydrogel qualitative gelation time	[122,123]
	UV spectroscopy	Hydrogel quantitative gelation time	[124]
	Rheometer	Hydrogel quantitative gelation time	[125]
	Gravimetric analysis	Hydrogel swelling ratio	[132]
		2. Hydrogel water-absorption capabilities	[131]
		3. Hydrogel degradation time/rate	[138,140]
	Tensiometer	How the incorporation of different components alters the surface properties of hydrogels	[135]
		2. Hydrogel hydrophilicity/hydrophobicity	[136]
		3. Hydrogel water-absorption capabilities	[126]
Cytocompatibility	Trypan blue assay	In vitro cytotoxicity of the hydrogel	[144,145]
	Live/dead assays		[147,148]
	MTT assay		[153]
	MTS assay		[158,159]
	XTT assay		[161,162]
	WST derivatives assays		[164,165]
	Alamar blue assay		[167,168]
